# B, N Codoped and Defect‐Rich Nanocarbon Material as a Metal‐Free Bifunctional Electrocatalyst for Oxygen Reduction and Evolution Reactions

**DOI:** 10.1002/advs.201800036

**Published:** 2018-04-24

**Authors:** Tao Sun, Jun Wang, Chuntian Qiu, Xiang Ling, Bingbing Tian, Wei Chen, Chenliang Su

**Affiliations:** ^1^ SZU‐NUS Collaborative Center and International Collaborative Laboratory of 2D Materials for Optoelectronic Science and Technology of Ministry of Education College of Optoelectronic Engineering Shenzhen University Shenzhen 518060 China; ^2^ Department of Chemistry National University of Singapore 3 Science Drive 3 Singapore 117543 Singapore; ^3^ Engineering Technology Research Center for 2D Material Information Function Devices and Systems of Guangdong Province Shenzhen University Shenzhen 518060 China

**Keywords:** boron and nitrogen codoping, carbon defects, metal‐free bifunctional electrocatalysts, oxygen evolution reactions, oxygen reduction reactions

## Abstract

The development of highly active, inexpensive, and stable bifunctional oxygen reduction reaction (ORR) and oxygen evolution reaction (OER) catalysts to replace noble metal Pt and RuO_2_ catalysts remains a considerable challenge for highly demanded reversible fuel cells and metal–air batteries. Here, a simple approach for the facile construction of a defective nanocarbon material is reported with B and N dopants (B,N‐carbon) as a superior bifunctional metal‐free catalyst for both ORR and OER. The catalyst is prepared by pyrolyzing the composites of ethyl cellulose and high‐boiling point 4‐(1‐naphthyl)benzeneboronic acid in NH_3_ atmosphere with an inexpensive Zn‐based template. The obtained porous B,N‐carbon with rich carbon defects exhibits excellent ORR and OER performances, including high activity and stability. In alkaline medium, B,N‐carbon material shows high ORR activity with an onset potential (*E*_onset_) reaching 0.98 V versus reversible hydrogen electrode (RHE), very close to that of Pt/C, a high electron transfer number and excellent stability. This catalyst also presents the admirable ORR activity in acidic medium with a high *E*_onset_ of 0.81 V versus RHE and a four‐electron process. The OER activity of B,N‐carbon is superior to that of the precious metal RuO_2_ and Pt/C catalysts. A Zn–air battery using B,N‐carbon as the air cathode exhibits a low voltage gap between charge and discharge and long‐term stability. The excellent electrocatalytic performance of this porous nanocarbon material is attributed to the combined positive effects of the abundant carbon defects and the heteroatom codopants.

## 1 Introduction

The development of clean and renewable energy technologies, such as fuel cells, metal–air batteries, and water splitting devices has attracted significant attention owing to the quickly growing energy demand and serious environmental crises.^1^, ^2^, ^3^, ^4^, ^5^ These technologies generally involve oxygen electrocatalysis, including the oxygen reduction reaction (ORR) and oxygen evolution reaction (OER), which directly determines the efficiency and applicability of the devices.^3^, ^4^, ^5^ Today, Ir/Ru‐ and Pt‐based materials are commonly used as the best OER and ORR catalysts, respectively. The large‐scale practical application of these renewable energy devices will be greatly hindered if the scarce and expensive noble metal‐based electrocatalysts cannot be replaced by other low‐cost and noble‐metal‐free yet efficient and durable electrodes.^4^, ^5^, ^6^, ^7^, ^8^ In addition, Ir/Ru‐ and Pt‐based materials alone do not effectively catalyze oxygen reduction and evolution simultaneously, while the rechargeable air‐based batteries require the combination of ORR and OER. Therefore, exploring bifunctional nonprecious metal (NPM) alternatives that are inexpensive, efficient, and stable is of particular significance today.

Since Dai and co‐workers' seminal report on the ORR in an alkaline medium using N‐doped carbon nanotubes as a new promising electrocatalyst in 2009,^9^ interest in carbon‐based metal‐free electrocatalysts, an important branch of NPM catalysts for electrocatalytic oxygen reactions, has dramatically increased due to their low cost and superb long‐term stability.^10^, ^11^ Since then, heteroatom dopants, such as N,^9^, ^12^, ^13^ B,^14^, ^15^ P,^16^, ^17^ and S,^18^, ^19^, ^20^ have been widely incorporated into nanocarbon materials to improve their electrocatalytic activities. The inherent differences in the electronegativity and atomic size between carbon and these heteroatoms could modify the inherent electronic structures of nanocarbon materials, thereby creating new catalytic sites and facilitating the chemisorption/desorption of intermediates and thus enhancing the catalytic activities.^9^, ^13^, ^14^, ^17^, ^21^, ^22^ In addition to heteroatom dopants, the intrinsic carbon defects, such as topological (pentagonal, heptagonal, etc.) and edge defects, of nanocarbon materials have also been demonstrated to play significant roles in the electrocatalytic oxygen reactions in both experimental and theoretical studies.^23^, ^24^, ^25^, ^26^, ^27^, ^28^, ^29^, ^30^, ^31^, ^32^ For example, Dai and co‐workers reported that the edge of graphite is much more active in the ORR than its basal plane, which was observed in situ using a self‐designed microdroplet electrochemical system.^23^ We recently found that the defective carbon nanocages exhibited excellent ORR activity, better than that of B‐doped carbon nanotubes and comparable to that of N‐doped nanocarbons. Density functional theory calculations indicated that the pentagon and zigzag edge defects in carbon nanocages were responsible for the high ORR activity.^24^ Inspired by all these results, an efficient approach to constructing superior carbon‐based electrocatalysts is likely the combination of specific heteroatom doping and engineered carbon defects due to their simultaneously positive effects.

Herein, we describe the development of a convenient strategy for the facile construction of porous B, N codoped nanocarbon (denoted B,N‐carbon) materials composed of the interconnected cuboidal hollow nanocages with fine graphitization and abundant carbon defects. This strategy was achieved via introducing ethyl cellulose (EC) and 4‐(1‐naphthyl)benzeneboronic acid (NBBA) onto the surface of a Zn‐based template prepared by a simple precipitation method, followed by pyrolysis in NH_3_ atmosphere at 800 °C and acid leaching, producing the desired B,N‐carbon material with multiple types of pores. Here, the abundant carbon defects were naturally formed by pyrolysis via the removal of the uniform oxygen species in EC and the introduction of B, N dopants into the carbon matrix. The decomposition of the Zn‐based template to ZnO with the release of CO_2_ and H_2_O during the heating process could also help to create micropores/carbon edge defects in the nanocarbon materials.^33^, ^34^, ^35^ The obtained nanocarbon material with a combination of B and N codopants and carbon defects is a highly reactive and durable electrocatalyst for both ORR and OER, which allows it to be an excellent air cathode exhibiting a low voltage gap between charge and discharge and a long lifetime in a homemade rechargeable Zn–air battery. The simple approach we utilized to construct the carbon‐based bifunctional electrocatalyst possesses overwhelming advantages over the current complicated preparation processes of bifunctional catalysts, especially those involving multistep codoping, hybridization, and defect incorporating.

## 2 Results and Discussion

**Figure 1:**
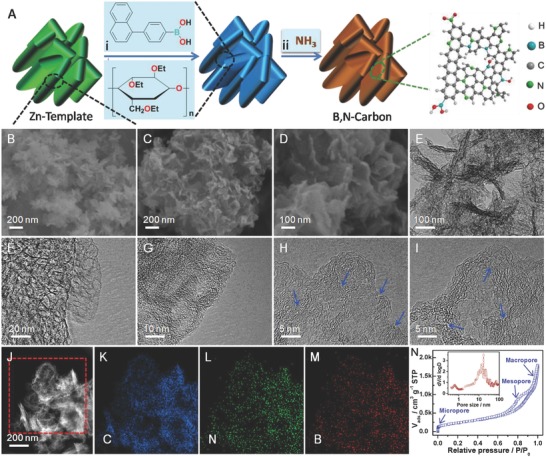
Schematic illustration of and characterizations on B,N‐carbon. A) Schematic illustration of the preparation process for B,N‐carbon: (i) uniform composites of the Zn‐based template, EC, and NBBA; (ii) pyrolysis and long‐time acid leaching to removal of ZnO template. B) Scanning electron microscopy (SEM) image of the Zn‐based template. C,D) SEM images of B,N‐carbon. D–H) TEM and HRTEM images of B,N‐carbon. I) High‐angle annular dark‐field TEM image. J) C mapping. K) N mapping. L) B mapping. M) N_2_ adsorption/desorption isotherms, and the inset is the pore size distribution.

A schematic illustration, typically morphological and structural properties of B,N‐carbon, is shown in **Figure** **1**. As described in the Experimental Section in the Supporting Information, Zn‐based templates prepared by a simple precipitation method are mixed with EC and NBBA under continuous stirring to form the homogeneous composites with uniform dispersion of EC and NBBA on the surface of template (step A). During the high‐temperature pyrolysis process, the well‐dispersed high‐boiling‐point B precursor facilitates the relatively uniform B doping in the carbon matrix. Carbon defects are created by the decomposition of the uniformly dispersed oxygen species in EC and Zn‐based template in release of CO*_x_* and H_2_O during the graphitization process. In addition, N dopants are also introduced by using NH_3_ as the carrier gas (step B). Figure 1B‐N shows the typically morphological and structural characterizations of the Zn‐based template and the defective B,N‐carbon. In this work, the morphological character of the Zn‐based template composed of the interconnected zinc carbonate hydroxide nanosheets is passed down to the nanocarbon (Figure 1B‐D and Figure S1, Supporting Information). The B,N‐carbon maintains the nanosheet morphology of the template with several hundred nanometers in size and 20–30 nm in thickness, which consists of the interconnected cuboidal hollow nanocages of ≈15–30 nm in size (Figure 1C‐G). High‐resolution transmission electron microscopy (HRTEM) images reveal the well‐graphitized carbon layers with a twisted morphology and some broken fringes in the shells (Figure 1H,I). The similar dispersions of C, B, and N species can be clearly observed by elemental mapping analysis (Figure 1J‐M). The N_2_ adsorption/desorption isotherm presents a typical IV‐type curve with two steep uptakes (*P/P*_0_ < 0.01, *P/P*_0_ > 0.97) and a hysteresis loop (0.40 < *P/P*_0_ < 0.90), indicating the coexistence of micropores (<2 nm), mesopores (2–50 nm), and macropores (>50 nm). The micropores mainly distribute around 0.6 nm in size, in accordance with the observed broken fringes in the graphitized shells (Figure 1H,I). By combining with HRTEM and N_2_ adsorption/desorption isotherm results, it is can be reasonably deduced that this carbon material possesses abundant carbon defects, that is, topological defects on the corners^24^ and edge defects on the broken fringes.^24^, ^28^, ^29^ This typically multistage pore distribution is beneficial for the diffusion of reactants during electrocatalytic reactions as well as creating more active sites exposed to the electrolyte.^36^, ^37^, ^38^ The specific surface area is calculated to be 849 m^2^ g^−1^ using the Brunauer–Emmett–Teller (BET) method. In addition, the main X‐ray diffraction peaks for the doped carbon materials shift to the high 2θ° relative to that of undoped one, which suggests that the dopants are successfully introduced into the carbon matrix, and the Zn‐based template can be easily removed through the acid washing which is also supported by the thermogravimetric analysis (Figures S2 and S3, Supporting Information).

**Figure 2:**
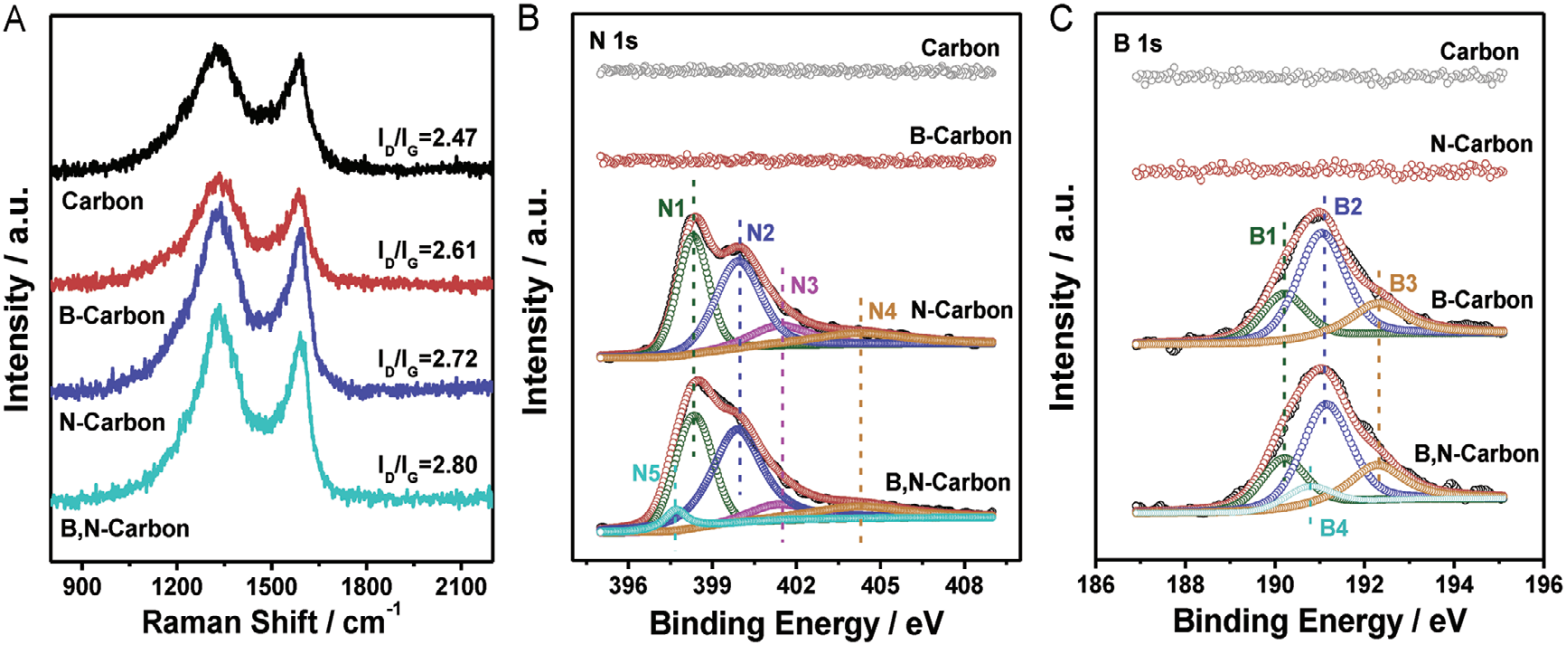
Raman and XPS spectra of carbon‐based metal‐free catalysts. A) Raman spectra. B) N 1s XPS spectra. C) B 1s XPS spectra.

The structures of carbon materials are further characterized by Raman and X‐ray photoelectron spectra (XPS), as shown in **Figure** **2**. Two obvious peaks at 1330 and 1590 cm^−1^ are identified in the Raman spectra, which correspond to the D‐band and G‐band of carbon, respectively. The G‐band arises from the stretching vibrations in the plane of sp^2^‐hybridized C atoms, whereas the D‐band is associated with the defects in the carbon structure. The ratio of the D‐band to G‐band reflects the defect level of carbon materials.^24^, ^27^ As shown in Figure 2A, the carbon materials doped with heteroatoms possess higher *I*_D_/*I*_G_ value than that of without doped one. B,N‐carbon has the highest *I*_D_/*I*_G_ value among the carbon materials, indicating that it has the highest ratio of carbon defects. The survey XPS results reveal the presence of the corresponding chemical elements in carbon materials except for B due to their low contents (1.08 at% B in B‐carbon, 1.22 at% B in B,N‐carbon) (Figure 2B,C and Figure S4, Supporting Information). High‐resolution XPS spectra are collected to further explore the bonding configuration, and the results are presented in Figure 2B,C and Table S1 (Supporting Information). The N 1s peak for N‐carbon can be deconvoluted into four contributions, that is, N1 (398.3 eV), N2 (399.9 eV), N3 (401.4 eV), and N4 (404.1 eV), which correspond to the pyridinic N, pyrrolic N, graphitic N, and oxidized N groups.^12^, ^17^, ^39^, ^40^, ^41^ A new peak observed at 397.6 eV for B,N‐carbon can be attributed to the N—B bond.^42^, ^43^ For B 1S, three B‐containing species can be clearly observed for B‐carbon, which are corresponded to the BC_3_ (190.2 eV), BC_2_O (191.1 eV), and BCO_2_ (192.3 eV) groups,^14^, ^15^, ^44^ while a new deconvoluted peak observed at 190.7 eV for B,N‐carbon is attributed to the B—N bond.^42^, ^43^, ^45^, ^46^, ^47^ Based on the above analyses, B and N heteroatoms are successfully introduced into the carbon matrix (Figure 2), which is also supported by the C 1s and O 1s spectra (Figure S5, Supporting Information).

**Figure 3:**
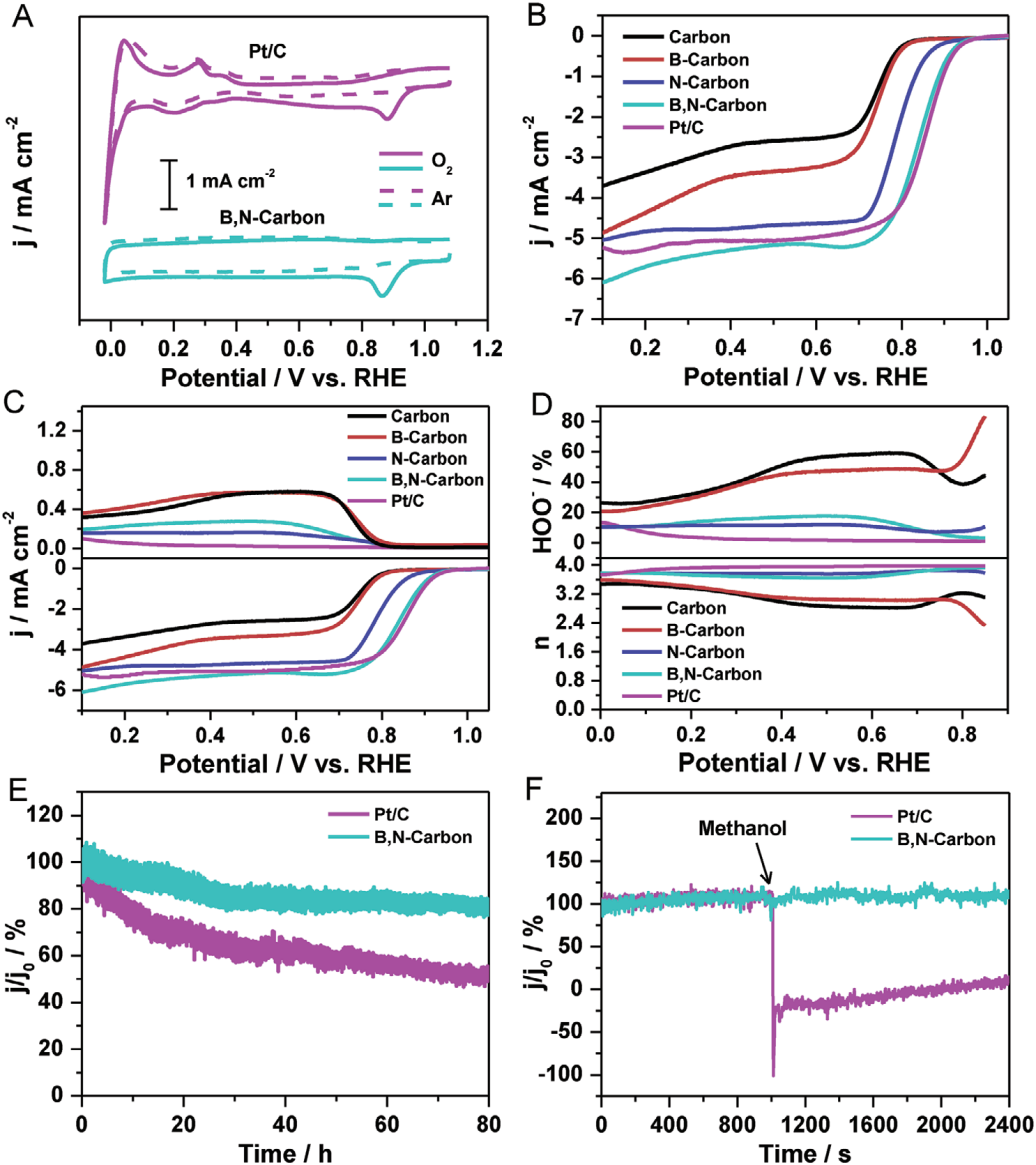
ORR performances of Pt/C (20 wt% Pt) and the series of carbon‐based metal‐free catalysts in 0.1 m KOH. A) CV curves in Ar‐saturated (‐ ‐ ‐) and O_2_‐saturated (**–**) KOH solutions. B) LSV curves (1600 rpm). C) RRDE curves (1600 rpm). D) Electron transfer number (*n*) and HOO^−^ yield versus potential. E) Stability test over 80 h. F) Methanol crossover test by addition of 2% (v/v) methanol to the electrochemical cell at 1000 s, as shown by an arrow. The tests in panels (E) and (F) were performed in O_2_‐saturated 0.1 m KOH under magnetic stirring. In panels (E) and (F), the bias voltage was set at −0.3 V versus Ag/AgCl, and *j*_0_ is the initial current.

The ORR performances of the as‐prepared carbon‐based metal‐free catalysts are evaluated using the electrochemical methods of the cyclic voltammetry (CV), linear scan voltammogram (LSV), rotating ring disk electrode (RRDE), and chronoamperometric response (CP), as shown in **Figure** **3**. In comparison with featureless case in the Ar‐saturated solution, an apparent ORR peak appears on the CV curve at 0.868 V versus reversible hydrogen electrode (RHE) for B,N‐carbon, a difference of only 15 mV from that of Pt/C at 0.882 V versus RHE. The onset potential (*E*_onset_) for B,N‐carbon is 0.98 V versus RHE, which is defined as the point at which the CV curves measured in the O_2_‐ and Ar‐saturated solutions separate from each other,^48^ and also supported by the LSV curve (Figure 3A and Figure S6, Supporting Information).^49^ The LSV curves confirm the excellent ORR activity of B,N‐carbon, which exhibits a half‐wave potential (*E*_1/2_) of 0.84 V versus RHE, a difference of only 17 mV from that of Pt/C, and a large current density that is much better than that of the B‐ or N‐doped ones and the pure one (Figure 3B). The electron transfer number per oxygen molecule (*n*) and the yield of corresponding peroxide species (HOO^−^) for ORR are determined from the RRDE curves (Figure 3C). It is clearly observed that *n* and HOO^−^ yield for B,N‐carbon are calculated to be 3.80 ± 0.15 and ≈12.8%, respectively, in the range of 0–0.85 V versus RHE, in good agreement with results from the Koutecky–Levich equations (Figure S7, Supporting Information). These results are better than those of the B‐doped and pure ones, similar to the N‐doped one (Figure 3D). In addition, B,N‐carbon presents an admirable stability in the long‐term test, only ≈18.8% decrease in the continuing 80 h test, much better than that of ≈48.3% decrease for Pt/C (Figure 3E). The *E*_onset_ for B,N‐carbon after 5000 cycles decreases by approximately only 10 mV, again indicating the excellent ORR stability in another evaluation system (Figure S8, Supporting Information). When 2% (v/v) methanol is added to the solution during the CP tests, the current for B,N‐carbon stays constant, while that for Pt/C suffers a sharp decrease (Figure 3F), indicating the excellent stability for methanol tolerance. The ORR performance of B,N‐carbon locates a top level in the reported carbon‐based metal‐free catalysts (Table S2, Supporting Information), exhibiting high activity, high electron transfer number, and superior stability. Finally, ORR activity in acidic medium for B,N‐carbon is also identified, which presents the splendid catalytic activity, including an *E*_onset_ of 0.81 V versus RHE and a 4e^−^ process with high *n* of 3.97 ± 0.01 and low H_2_O_2_ yield of below 2.5% in the range of 0.10–0.70 V versus RHE (Figure S9, Supporting Information).

**Figure 4:**
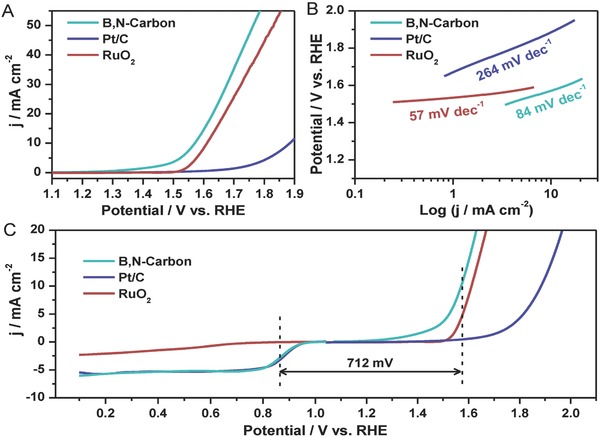
OER performances of the series of catalysts. A) LSV curves measured at a rotation speed of 1600 rpm with a scan rate of 5 mV s^−1^. B) Tafel plots. C) Bifunctional catalytic activities for ORR and OER in O_2_‐saturated 1.0 m KOH.

To reveal the OER performance of B,N‐carbon, ring disk electrodes modified by carbon‐based materials as working electrodes are collected in 1.0 m KOH solution using a three‐electrode system, as shown in **Figure** **4**. B,N‐carbon presents the outstanding OER activity, better than that of precious metal catalysts (RuO_2_ and Pt/C). Specifically, the potential at 10 mA cm^−2^ (*E_j_*_  = 10_) for B,N‐carbon, RuO_2_, and Pt/C are 1.57, 1.61, and 1.89 V versus RHE, respectively. Clearly, B,N‐carbon can efficiently catalyze OER with the low overpotential, which is better than those of other carbon‐based metal‐free catalysts and also comparable to those of the previously reported OER catalysts (Figure S10 and Table S3, Supporting Information). Tafel slope of B,N‐carbon is 84 mV dec^−1^, locating at a good level in the reported carbon‐based metal‐free catalysts (Table S3, Supporting Information),^17^, ^19^, ^25^, ^26^, ^50^, ^51^, ^52^ indicating that B,N‐carbon exhibits the outstanding electrocatalytic reaction kinetics. The good stability for B,N‐carbon is confirmed by the LSV curves after 1000 cycles tests (Figure S11, Supporting Information). In addition, the electrochemically active surface areas of B,N‐carbon, Pt/C, and RuO_2_ are also collected, which is another important factor to study the characteristic of electrocatalysts.^53^ B,N‐carbon presents the highest double‐layer capacitance (*C*_dl_) (22 mF cm^−2^) compared with the Pt/C (7 mF cm^−2^) and RuO_2_ (5 mF cm^−2^), which indicates the more exposed surface area in electrolyte (Figure S12, Supporting Information). The overall oxygen activities of catalysts can be evaluated by the value of Δ*E*,^4^ which is defined as the potential difference between *E_j_*_  = 10_ (OER) and *E*_1/2_ (ORR), namely Δ*E* = *E_j_*_  = 10_ – *E*_1/2_. A lower Δ*E* value represents better bifunctional catalytic activity of an electrocatalyst for ORR and OER, which is a very important parameter for evaluating the efficiency of catalysts.^4^, ^54^ The Δ*E* of B,N‐carbon is calculated to be 0.712 V, lower than those of the reported nonprecious metal catalysts (e.g., N‐doped carbon, Δ*E* = 0.90 V;^25^ S and N codoped graphitic carbon, Δ*E* = 0.77 V;^55^ P and N codoped graphene/carbon, Δ*E* = 0.71 V;^56^ CuCoO*_x_*/FeOOH, Δ*E* = 0.72 V;^53^ Mn*_x_*O*_y_*, Δ*E* = 0.87 V;^54^ Ni_3_Fe‐N‐doped carbon, Δ*E* = 0.84 V;^57^ and Fe_0.5_Co_0.5_O*_x_*, Δ*E* = 0.78 V.^58^), confirming the high bifunctional activity of B,N‐carbon for OER and ORR simultaneously.

To reveal the reasons for the excellent electrocatalytic performance of B,N‐carbon in ORR and OER, we also prepared another pure carbon material using the poly(methyl methacrylate) (PMMA) as the C source to replace EC. Clearly, the content ratio of O species in EC is higher than that of PMMA and uniformly dispersed in its skeleton, thus leading to more carbon defects following good dispersion in the final carbon material owing to the reaction of O species with C species at high temperature (here, 800 °C). Carbon defects play a significant contribution in improving the electrocatalytic reactions for carbon materials.^24^, ^25^, ^26^, ^27^ It can be clearly observed that pure carbon (carbon‐PMMA) prepared by using PMMA as C source shows the inferior ORR and OER activities than that of one (carbon‐EC) using EC as C source (Figures S13 and S14, Supporting Information). From combination of the BET‐specific surface area with the Raman results for two carbon materials, we can know that these two materials possess the similar N_2_ adsorption/desorption isotherms and comparable specific surface area, and only difference exists in the of *I*_D_/*I*_G_ values (carbon‐EC versus carbon‐PMMA: 2.47 versus 2.25) determined from Raman analysis (Figure S15, Supporting Information). Therefore, the high ORR and OER activities can be attributed to the presence of more carbon defects in carbon‐EC. In a short summary, the high density of carbon defects could help to activate the graphitic π‐electron system^6^, ^14^, ^22^ and facilitate the adsorption of reactants. These B, N codopants not only synergistically modulate the charge density of carbon materials, but also create new catalytic sites due to their different electron properties and atom sizes. All these positive effects co‐contribute to the excellent performance of the defective B,N‐carbon.

**Figure 5:**
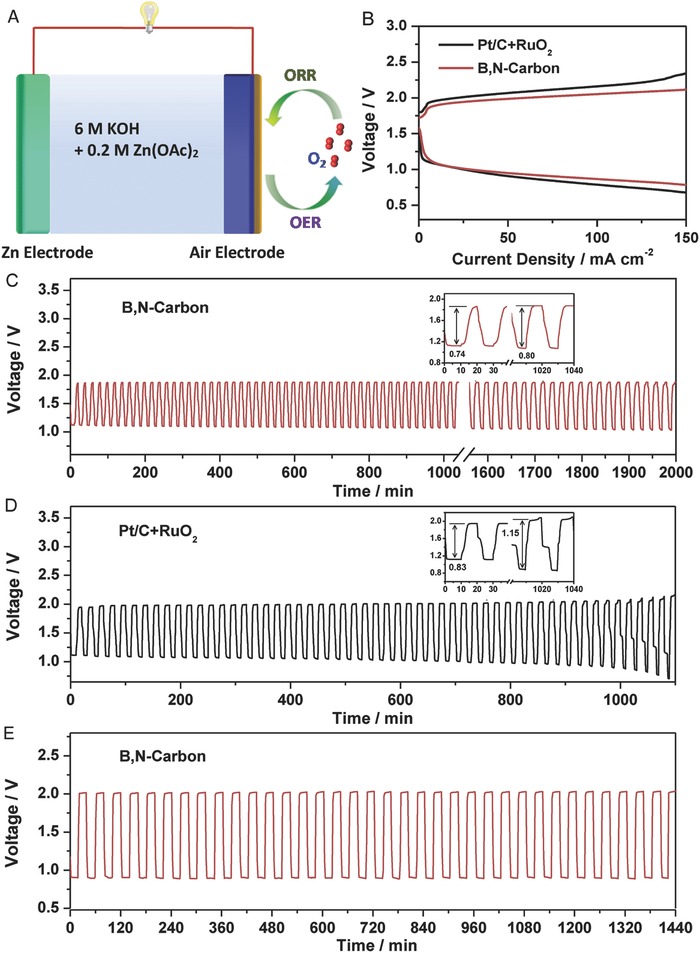
Electrochemical performances of the Zn–air batteries using B,N‐carbon and Pt/C+RuO_2_ catalysts. A) Illustration of the Zn–air battery. B) Charge and discharge polarization curves of two‐electrode rechargeable Zn–air batteries. C) Galvanostatic charge–discharge cycling obtained at 5 mA cm^−2^ for B,N‐carbon catalyst. D) Galvanostatic charge–discharge cycling obtained at 5 mA cm^−2^ for Pt/C+RuO_2_ catalyst. E) Cycling performance at the charging and discharging current density of 50 mA cm^−2^.

Finally, a Zn–air battery using B,N‐carbon as an air cathode and Zn plate as anode is built to evaluate the practically electrocatalytic performance in the application of metal–air batteries, as shown in **Figure** **5** (Figure S16, Supporting Information). The discharge and charge polarization curves for Zn–air battery with B,N‐carbon as air cathode present a lower voltage gap than that of Pr/C+RuO_2_ catalyst. Compared to the battery constructed with Pt/C+RuO_2_ catalyst, an obviously better charging and discharging performance is observed for the battery using B,N‐carbon. When cycled at a current density of 5 mA cm^−2^, the battery using the B,N‐carbon electrode exhibits the charge and discharge potentials of 1.86 and 1.12 V, respectively, presenting a small overpotential of 0.74 V (Figure 5C). At a current density of 100 mA cm^−2^, the power densities of B,N‐carbon and Pr/C+RuO_2_ catalyst are 143 and 129 mW cm^−2^, respectively (Figure S17, Supporting Information). The internal electric resistance of the rechargeable Zn–air battery with B,N‐carbon is 4.0 Ω, much lower than that of the Zn‐air battery with Pt/C+RuO_2_ (9.6 Ω) (Figure S18, Supporting Information). Meanwhile, the battery using the B,N‐carbon electrode exhibits a better stability and lower overpotential than that of using Pt/C+RuO_2_ catalyst. Specifically, the overpotential of battery using Pt/C and RuO_2_ shifts to 1.15 V from the initial one of 0.83 V after 50 cycles, obviously higher than that of B,N‐carbon (initial, 0.74 vs 0.80 V, after 50 cycles). A high applied current density for Zn–air battery can increase its corresponding overpotential and cause deterioration of catalyst. As shown in Figure 5E, the Zn–air battery using B,N‐carbon as electrode exhibits an excellent stability even at a high current density of 50 mA cm^−2^, in which nearly constants between charging and discharging voltages can be observed. Clearly, the developed B,N‐carbon catalyst not only possesses the high intrinsic catalytic activity and stability, but also enables Zn–air battery with high cell efficiency and long cycle life.

## 3 Conclusions

In summary, we have demonstrated that a porous B, N codoped nanocarbon, being composed of the interconnected cuboidal hollow nanocages with abundant carbon defects and fine graphitization, is a highly active and stable metal‐free bifunctional electrocatalyst for ORR and OER. This B,N‐carbon material with multiple types of pores performs a high ORR activity comparable to that of Pt/C and superb stability, as well as excellent OER activity better than that of Pt/C and RuO_2_. The excellent electrocatalytic performance is attributed to the combined positive effects of heteroatom dopants and carbon defects. Impressively, Zn–air battery using the B,N‐carbon as the air cathode presents a low voltage gap between charge and discharge, and better long‐term stability in comparison to the noble metal Pt/C+RuO_2_ catalyst. This work provides a simple and efficient approach to develop the advanced carbon‐based metal‐free electrocatalysts by utilizing the precursors to do both heteroatoms doping and creating carbon defects simultaneously.

## Conflict of Interest

The authors declare no conflict of interest.

## Supporting information

Supplementary

## References

[1] R. Borup , J. Meyers , B. Pivovar , Y. S. Kim , R. Mukundan , N. Garland , D. Myers , M. Wilson , F. Garzon , D. Wood , P. Zelenay , K. More , K. Stroh , T. Zawodzinski , J. Boncella , J. E. McGrath , M. Inaba , K. Miyatake , M. Hori , K. Ota , Z. Ogumi , S. Miyata , A. Nishikata , Z. Siroma , Y. Uchimoto , K. Yasuda , K. Kimijima , N. Iwashita , Chem. Rev. 2007, 107, 3904. 17850115 10.1021/cr050182l

[2] C. G. Morales‐Guio , L. A. Stern , X. L. Hu , Chem. Soc. Rev. 2014, 43, 6555. 24626338 10.1039/c3cs60468c

[3] M. K. Debe , Nature 2012, 486, 43. 22678278 10.1038/nature11115

[4] Y. Jiao , Y. Zheng , M. Jaroniec , S. Z. Qiao , Chem. Soc. Rev. 2015, 44, 2060. 25672249 10.1039/c4cs00470a

[5] J. S. Lee , S. T. Kim , R. G. Cao , N. S. Choi , M. L. Liu , K. T. Lee , J. Cho , Adv. Energy Mater. 2011, 1, 34.

[6] H. A. Gasteiger , N. M. Markovic , Science 2009, 324, 48. 19342578 10.1126/science.1172083

[7] J. Li , G. F. Zheng , Adv. Sci. 2017, 4, 1600380. 10.1002/advs.201600380 PMC5357991 28331791

[8] T. Sun , B. B. Tian , J. Lu , C. L. Su , J. Mater. Chem. A 2017, 5, 18933.

[9] K. P. Gong , F. Du , Z. H. Xia , M. Durstock , L. M. Dai , Science 2009, 323, 760. 19197058 10.1126/science.1168049

[10] X. E. Liu , L. M. Dai , Nat. Rev. Mater. 2016, 1, 16064.

[11] J. P. Paraknowitsch , A. Thomas , Energy Environ. Sci. 2013, 6, 2839.

[12] S. Chen , J. Y. Bi , Y. Zhao , L. J. Yang , C. Zhang , Y. W. Ma , Q. Wu , X. Z. Wang , Z. Hu , Adv. Mater. 2012, 24, 5593. 22899547 10.1002/adma.201202424

[13] G. L. Chai , K. P. Qiu , M. Qiao , M. M. Titirici , C. X. Shang , Z. X. Guo , Energy Environ. Sci. 2017, 10, 1186.

[14] L. J. Yang , S. J. Jiang , Y. Zhao , L. Zhu , S. Chen , X. Z. Wang , Q. Wu , J. Ma , Y. W. Ma , Z. Hu , Angew. Chem., Int. Ed. 2011, 50, 7132. 10.1002/anie.201101287 21688363

[15] T. V. Vineesh , M. P. Kumar , C. Takahashi , G. Kalita , S. Alwarappan , D. K. Pattanayak , T. N. Narayanan , Adv. Energy Mater. 2015, 5, 1500658.

[16] Z. W. Liu , F. Peng , H. J. Wang , H. Yu , W. X. Zheng , J. Yang , Angew. Chem., Int. Ed. 2011, 50, 3257. 10.1002/anie.201006768 21381161

[17] J. T. Zhang , Z. H. Zhao , Z. H. Xia , L. M. Dai , Nat. Nanotechnol. 2015, 10, 444. 25849787 10.1038/nnano.2015.48

[18] Z. Yang , Z. Yao , G. F. Li , G. Y. Fang , H. G. Nie , Z. Liu , X. M. Zhou , X. A. Chen , S. M. Huang , ACS Nano 2012, 6, 205. 22201338 10.1021/nn203393d

[19] A. M. El‐Sawy , I. M. Mosa , D. Su , C. J. Guild , S. Khalid , R. Joesten , J. F. Rusling , S. L. Suib , Adv. Energy Mater. 2016, 6, 1501966.

[20] T. Sun , Q. Wu , Y. F. Jiang , Z. Q. Zhang , L. Y. Du , L. J. Yang , X. Z. Wang , Z. Hu , Chem. ‐ Eur. J. 2016, 22, 10326. 27150558 10.1002/chem.201601535

[21] C. G. Hu , L. M. Dai , Angew. Chem., Int. Ed. 2016, 55, 11736. 10.1002/anie.201509982 27460826

[22] D. W. Wang , D. S. Su , Energy Environ. Sci. 2014, 7, 576.

[23] A. L. Shen , Y. Q. Zou , Q. Wang , R. A. W. Dryfe , X. B. Huang , S. Dou , L. M. Dai , S. Y. Wang , Angew. Chem., Int. Ed. 2014, 53, 10804. 10.1002/anie.201406695 25124986

[24] Y. F. Jiang , L. J. Yang , T. Sun , J. Zhao , Z. Y. Lyu , O. Zhuo , X. Z. Wang , Q. Wu , J. Ma , Z. Hu , ACS Catal. 2015, 5, 6707.

[25] C. Tang , H. F. Wang , X. Chen , B. Q. Li , T. Z. Hou , B. S. Zhang , Q. Zhang , M. M. Titirici , F. Wei , Adv. Mater. 2016, 28, 6845. 27167616 10.1002/adma.201601406

[26] Y. Jia , L. Z. Zhang , A. J. Du , G. P. Gao , J. Chen , X. C. Yan , C. L. Brown , X. D. Yao , Adv. Mater. 2016, 28, 9532. 27622869 10.1002/adma.201602912

[27] Z. J. Liu , Z. H. Zhao , Y. Y. Wang , S. Dou , D. F. Yan , D. D. Liu , Z. H. Xia , S. Y. Wang , Adv. Mater. 2017, 29, 1606207.

[28] S. T. Senthilkumar , S. O. Park , J. Kim , S. M. Hwang , S. K. Kwak , Y. Kim , J. Mater. Chem. A 2017, 5, 14174.

[29] L. Tao , Q. Wang , S. Dou , Z. L. Ma , J. Huo , S. Y. Wang , L. M. Dai , Chem. Commun. 2016, 52, 2764. 10.1039/c5cc09173j 26757794

[30] C. Tang , Q. Zhang , Adv. Mater. 2017, 29, 1604103. 10.1002/adma.201604103 28067956

[31] D. F. Yan , Y. X. Li , J. Huo , R. Chen , L. M. Dai , S. Y. Wang , Adv. Mater. 2017, 29, 1606459. 10.1002/adma.201606459 28508469

[32] M. T. Li , L. P. Zhang , Q. Xu , J. B. Niu , Z. H. Xia , J. Catal. 2014, 314, 66.

[33] P. Q. Yin , T. Yao , Y. E. Wu , L. R. Zheng , Y. Lin , W. Liu , H. X. Ju , J. F. Zhu , X. Hong , Z. X. Deng , G. Zhou , S. Q. Wei , Y. D. Li , Angew. Chem., Int. Ed. 2016, 55, 10800. 10.1002/anie.201604802 27491018

[34] S. G. Wang , Z. T. Cui , J. W. Qin , M. H. Cao , Nano Res. 2016, 9, 2270.

[35] P. Strubel , S. Thieme , T. Biemelt , A. Helmer , M. Oschatz , J. Brückner , H. Althues , S. Kaskel , Adv. Funct. Mater. 2015, 25, 287.

[36] J. Liang , R. F. Zhou , X. M. Chen , Y. H. Tang , S. Z. Qiao , Adv. Mater. 2014, 26, 6074. 25042569 10.1002/adma.201401848

[37] T. Sun , Q. Wu , O. Zhuo , Y. F. Jiang , Y. F. Bu , L. J. Yang , X. Z. Wang , Z. Hu , Nanoscale 2016, 8, 8480. 27055582 10.1039/c6nr00760k

[38] Q. Li , T. Y. Wang , D. Havas , H. G. Zhang , P. Xu , J. T. Han , J. Cho , G. Wu , Adv. Sci. 2016, 3, 1600140. 10.1002/advs.201600140 PMC5102660 27980990

[39] F. Jaouen , J. Herranz , M. Lefevre , J. P. Dodelet , U. I. Kramm , I. Herrmann , P. Bogdanoff , J. Maruyama , T. Nagaoka , A. Garsuch , J. R. Dahn , T. Olson , S. Pylypenko , P. Atanassov , E. A. Ustinov , ACS Appl. Mater. Interfaces 2009, 1, 1623. 20355776 10.1021/am900219g

[40] W. Ding , Z. D. Wei , S. G. Chen , X. Q. Qi , T. Yang , J. S. Hu , D. Wang , L. J. Wan , S. F. Alvi , L. Li , Angew. Chem., Int. Ed. 2013, 52, 11755. 10.1002/anie.201303924 24038758

[41] G. J. He , M. Qiao , W. Y. Li , Y. Lu , T. T. Zhao , R. J. Zou , B. Li , J. A. Darr , J. Q. Hu , M. M. Titirici , I. P. Parkin , Adv. Sci. 2017, 4, 1600214. 10.1002/advs.201600214 PMC5238742 28105397

[42] S. Y. Kim , J. Park , H. C. Choi , J. P. Ahn , J. Q. Hou , H. S. Kang , J. Am. Chem. Soc. 2007, 129, 1705. 17243688 10.1021/ja067592r

[43] L. Song , L. J. Ci , H. Lu , P. B. Sorokin , C. H. Jin , J. Ni , A. G. Kvashnin , D. G. Kvashnin , J. Lou , B. I. Yakobson , P. M. Ajayan , Nano Lett. 2010, 10, 3209. 20698639 10.1021/nl1022139

[44] Z. S. Wu , W. C. Ren , L. Xu , F. Li , H. M. Cheng , ACS Nano 2011, 5, 5463. 21696205 10.1021/nn2006249

[45] T. W. Lin , C. Y. Su , X. Q. Zhang , W. J. Zhang , Y. H. Lee , C. W. Chu , H. Y. Lin , M. T. Chang , F. R. Chen , L. J. Li , Small 2012, 8, 1384. 22378619 10.1002/smll.201101927

[46] Y. Zhao , L. J. Yang , S. Chen , X. Z. Wang , Y. W. Ma , Q. Wu , Y. F. Jiang , W. J. Qian , Z. Hu , J. Am. Chem. Soc. 2013, 135, 1201. 23317479 10.1021/ja310566z

[47] L. J. Ci , L. Song , C. H. Jin , D. Jariwala , D. X. Wu , Y. J. Li , A. Srivastava , Z. F. Wang , K. Storr , L. Balicas , F. Liu , P. M. Ajayan , Nat. Mater. 2010, 9, 430. 20190771 10.1038/nmat2711

[48] T. Sun , Q. Wu , R. C. Che , Y. F. Bu , Y. F. Jiang , Y. Li , L. J. Yang , X. Z. Wang , Z. Hu , ACS Catal. 2015, 5, 1857.

[49] J. C. Li , P. X. Hou , S. Y. Zhao , C. Liu , D. M. Tang , M. Cheng , F. Zhang , H. M. Cheng , Energy Environ. Sci. 2016, 9, 3079.

[50] L. Q. Li , H. B. Yang , J. W. Miao , L. P. Zhang , H. Y. Wang , Z. P. Zeng , W. Huang , X. C. Dong , B. Liu , ACS Energy Lett. 2017, 2, 294.

[51] H. B. Yang , J. W. Miao , S. F. Hung , J. Z. Chen , H. B. Tao , X. Z. Wang , L. P. Zhang , R. Chen , J. J. Gao , H. M. Chen , L. M. Dai , B. Liu , Sci. Adv. 2016, 2, e1501122. 27152333 10.1126/sciadv.1501122 PMC4846433

[52] D. Q. Li , B. W. Ren , Q. Y. Jin , H. Cui , C. X. Wang , J. Mater. Chem. A 2018, 6, 2176.

[53] M. Kuang , Q. H. Wang , H. T. Ge , P. Han , Z. X. Gu , A. M. Al‐Enizi , G. F. Zheng , ACS Energy Lett. 2017, 2, 2498.

[54] J. Masa , W. Xia , I. Sinev , A. Q. Zhao , Z. Y. Sun , S. Grützke , P. Weide , M. Muhler , W. Schuhmann , Angew. Chem., Int. Ed. 2014, 53, 8508. 10.1002/anie.201402710 24975388

[55] C. G. Hu , L. M. Dai , Adv. Mater. 2017, 29, 1604942.

[56] R. Li , Z. D. Wei , X. L. Gou , ACS Catal. 2015, 5, 4133.

[57] G. T. Fu , Z. M. Cui , Y. F. Chen , Y. T. Li , Y. W. Tang , J. B. Goodenough , Adv. Energy Mater. 2016, 6, 1601172.

[58] L. Wei , H. E. Karahan , S. L. Zhai , H. W. Liu , X. C. Chen , Z. Zhou , Y. J. Lei , Z. W. Liu , Y. Chen , Adv. Mater. 2017, 29, 1701410. 10.1002/adma.201701410 28804931

