# Supplementary material for: B, N Codoped and Defect‐Rich Nanocarbon Material as a Metal‐Free Bifunctional Electrocatalyst for Oxygen Reduction and Evolution Reactions
Source: Adv Sci (Weinh). 2018 Apr 24;5(7):1800036. doi: 10.1002/advs.201800036 (PMC6051395; doi:10.1002/advs.201800036)
Supplement: Supplementary file 1 — Supplementary [file ADVS-5-1800036-s001.pdf]

Copyright WILEY-VCH Verlag GmbH & Co. KGaA, 69469 Weinheim, Germany, 2018.

Supporting Information

for *Adv. Sci.,* DOI: 10.1002/advs.201800036

B, N Codoped and Defect-Rich Nanocarbon Material as a Metal-Free Bifunctional Electrocatalyst for Oxygen Reduction and Evolution Reactions

*Tao Sun, Jun Wang, Chuntian Qiu, Xiang Ling, Bingbing Tian, Wei Chen, and Chenliang Su**

Copyright WILEY-VCH Verlag GmbH & Co. KGaA, 69469 Weinheim, Germany, 2016.

Supporting Information

B, N Co-doped and Defect-rich Nanocarbon Material as a Metal-free Bifunctional Electrocatalyst for Oxygen Reduction and Evolution Reactions

*Tao Sun, Jun Wang, Chuntian Qiu, Xiang Ling, Bingbing Tian, Wei Chen, and Chenliang Su**

Dr. T. Sun, Dr. J. Wang, Dr. C. T. Qiu, Dr. X. Ling, Dr. B. B. Tian, Prof. C. L. Su,
SZU-NUS Collaborative Center and International Collaborative Laboratory of 2D Materials for Optoelectronic Science & Technology of Ministry of Education, College of Optoelectronic Engineering, Shenzhen University, Shenzhen 518060, China
E-mail: chmsuc@szu.edu.cn
Dr. T. Sun, Dr. B. B. Tian, Prof. W. Chen
Department of Chemistry, National University of Singapore, 3 Science Drive 3, Singapore 117543
Dr. C. T. Qiu, Prof. C. L. Su,
Engineering Technology Research Center for 2D Material Information Function Devices and Systems of Guangdong Province, Shenzhen University, Shenzhen 518060, China

Context:

Experimental section

Figure S1. SEM image (A) and XRD pattern (B) of Zn-based template.

Figure S2. (A) XRD patterns of catalysts. (B) XRD patterns for the B,N-Carbon before and after the removal of ZnO template.

Figure S3. TG and DTG curves for B,N-Carbon.

Figure S4. Full (A) and partial enlarged (B) XPS spectra of carbon-based catalysts.

Figure S5. C 1s (A) and O 1s (B) spectra of carbon-based metal-free catalysts.

Figure S6. LSV curve of B,N-Carbon in O_2-saturated 0.1 M KOH at 1600 rpm

Figure S7. LSV curves (A, C, E, G and I) at various rotation speeds and K-L plots (B, D, F, H and J) of catalysts in O_2-saturated 0.1 M KOH.

Figure S8. LSV curves of B,N-Carbon catalyst before and after 5000 cycles in O_2-saturated 0.1 M KOH.

Figure S9. (A) CV curves of B,N-Carbon in O_2- and Ar-saturated 0.5 M H_2SO_4. (B) LSV curves of catalysts in O_2-saturated 0.5 M H_2SO_4. (C) RRDE curve of B,N-Carbon. (D) *n* and H_2O_2 yield of B,N-Carbon at different potentials.

Figure S10. LSV curves of the series of carbon-based metal-free catalysts in O_2-saturated 1.0 M KOH.

Figure S11. LSV curves of B,N-Carbon catalyst before and after 1000 cycles in O_2-saturated 1.0 M KOH.

Figure S12. (A-C) CV curves at various scan rates of (A) B,N-Carbon, (B) Pt/C, (C) RuO_2, (D) Charging current density differences ($\Delta j = j_a - j_c$) plotted against scan rates of B,N-Carbon, Pt/C, and RuO_2. The linear slope is equivalent to twice of the double-layer capacitance C_{dl}.

Figure S13. CV (A) and LSV (B) curves of carbon prepared by using different carbon-containing precursors. (C) RRDE curve of carbon prepared by using the PMMA as the precursor. (D) Electron transfer number (*n*) and HOO^- yield calculated from RRDE curve.

Figure S14. LSV curves of carbon prepared by using different carbon-containing precursors.

Figure S15. Raman spectra (A) and N_2 adsorption/desorption isotherms (B) of carbon prepared by using different carbon-containing precursors.

Figure S16. (A) Photographs of the rechargeable Zn-air battery. (B-E) Photographs of the rechargeable Zn-air battery recorded from the different directions and the pump.

Figure S17. Power density curves of the primary Zn-air batteries using Pt/C+RuO_2 and B,N-Carbon catalysts.

Figure S18. EIS spectra of the rechargeable Zn-air batteries with B,N-Carbon or Pt/C+RuO_2 catalysts.

Table S1. Element contents detected by XPS.

Table S2. Summary of ORR performance for carbon-based metal-free catalysts in alkaline medium.

Table S3. Summary of OER performance for carbon-based metal-free catalysts in alkaline medium.

References

1. Experimental section

1.1 Synthesis of Zn-based template

All chemicals are analytical reagent without further purification. Zn-based template was prepared via a precipitation method. $ZnCl_2$ (0.04 mol) was dissolved in 400 ml distilled water with containing 0.5 ml HCl (36-38 wt%) under stirring. Then 100 ml $NaHCO_3$ (0.08 mol) solution was slowly dropped into the Zn-based solution for 4 h. Finally, the mixture was stirring for 24 h, filtrating and drying at 60 ^{o}C for 12 h. The resulting white powder is the Zn-based template.

1.2 Synthesis of B,N-Carbon material

In a typical procedure, 4.0 g ethyl cellulose (EC) and 0.4 g 4-(1-naphthyl)benzeneboronic acid (NBBA) were dissolved in 150 ml acetone. Then 3.0 g Zn-based template was added into the above mixture and stirring for 12 h. After volatilizing acetone at room temperature under stirring, the product was placed into the tube furnace and ramped to 800 °C at 10 °C min^{-1} in a NH_3 flow. The furnace was cooled naturally to room temperature after maintaining it at 800 °C for 1 h. The as-prepared sample was treated with 4 mol L^{-1} (4 M) hydrochloric acid solution for continuing stirring 48 h and repeatedly flushed with deionized water for the removal of the ZnO template, finally vacuum drying at 60 °C for 12 h. The resulting sample is B and N co-doped nanocarbon material, and renamed as B,N-Carbon. In this process, B doped carbon (B-Carbon), N doped carbon (N-Carbon) and pure carbon (Carbon or Carbon-EC) are obtained by the same ways just pyrolyzing in Ar flow, without the presence of NBBA, and pyrolyzing in Ar flow without the presence of NBBA, respectively. When EC is replaced by ploy(methyl methacrylate) (PMMA, 3 g), the Carbon-PMMA sample can be obtained which is another pure carbon material without dopants.

1.3 Characterization

The catalysts were characterized by the field emission scanning electron microscopy (FESEM, JSM-7800F), X-ray diffraction (XRD, Ultima Ⅳ, X-ray diffractometer with Cu K_{α}), Raman spectroscopy (LabRAM Aramis, laser excitation at 532 nm), transmission electron microscopy (TEM) (FEI Tecnai G2 F30, operating at 300 kV) and X-ray photoelectron spectroscopy (XPS, Thermo ESCALAB 250). The binding energies of XPS spectra referred to C 1s at 284.6 eV. Thermogravimetry analysis (TG, Netzsch STA-449F3) was conducted at a heating rate of 10 °C min^{-1} under 8 % O_2/Ar atmosphere. N_2 adsorption/desorption isotherms were measured on Thermo Fisher Scientifc Surfer Gas Adsorption Porosimeter at

77 K. The specific surface area was calculated from the adsorption branch of N_2 isotherm with BET method in the linear relative pressure (P/P_0) range of 0.05-0.3. The pore size distribution and pore volume were calculated from Horvath-Kawazoe method for micropores, and Barrett-Joyner-Halenda method for mesopores and macropores.

1.4 Electrochemical measurements

The electrochemical tests including cyclic voltammetry (CV) and rotating disk electrode (RDE) and rotating ring disk electrode (RRDE) voltammetry were performed on an MSR electrode rotator (Pine Instrument Co.) coupled with a CHI 760E workstation (CH Instruments) with the counter electrode of Pt wire and the reference electrode of Ag/AgCl (3 M KCl).

ORR tests: Briefly, the catalyst inks were prepared by adding 3 mg of carbon-based catalysts to a mixture of 0.80 mL of deionized water, 0.20 mL of ethanol, and 50 μL of Nafion (Dupont, 5 wt %) with 1 h ultrasonic treatment. A 10 μL portion of fresh catalyst ink was dropped onto a glassy-carbon electrode (GC; 0.196 cm^2, Pine Instrument Co.) and dried at room temperature for 12 h. Thus, the catalyst loading on the GC electrodes was 0.146 mg cm^{-2}. The commercial Pt/C (20 wt % Pt) catalyst on the electrode was 20 μg_{Pt} cm^{-2}. The CV, RDE, and RRDE curves were recorded in O_2-saturated 0.1 M KOH (or 0.5 M H_2SO_4) at a scan rate of 10 mV s^{-1}, and the Pt ring electrode was polarized at 0.5 V vs. Ag/AgCl in KOH (or 1.0 V vs. Ag/AgCl in H_2SO_4). The ORR stability of B, N-Carbon and Pt/C catalysts was measured by the chronoamperometric (CP) response at -0.3 V vs. Ag/AgCl in the O_2-saturated KOH electrolyte by constantly bubbling O_2 (15 mL min^{-1}) with magnetic stirring during the whole process. Similarly, in the methanol crossover tests, 2% (v/v) methanol was introduced into O_2-saturated electrolyte at 1000 s. In addition, another method to evaluate the stability of B,N-Carbon was measured, i.e., RDE curve for B,N-Carbon was also collected after 5000 cycles in CV with the scan rate of 100 mV s^{-1} between 0.1 and -1.0 V vs. Ag/AgCl in the O_2-saturated KOH electrolyte by constantly bubbling O_2.

The electron transfer number (*n*) peroxide species (HOO^- or H_2O_2) percentage can be calculated by the following equations based on RRED:

$$n = 4 \times \frac{i_d}{i_r / N + i_d} \qquad (1)$$

$$\% \text{ peroxide} = 200 \times \frac{i_r / N}{i_r / N + i_d} \qquad (2)$$

Where i_d is disk current, i_r is ring current and N is the current collection efficiency of the Pt ring (N = 0.37).

The electron transfer number is also calculated by the Koutecky-Levich (K-L) equation based on RDE data:

$$\frac{1}{j}=\frac{1}{j_L}+\frac{1}{j_K}=\frac{1}{B\omega^{1/2}}+\frac{1}{j_K} \qquad (3)$$

$$B=0.62\mathrm{n}FC_0(D_0)^{2/3}v^{-1/6} \qquad (4)$$

$$j_K=nFkC_0 \qquad (5)$$

Where j is the measured current density, j_K and j_L are the kinetic- and diffusion-limiting current density, ω is the electrode rotating rate, F is the Faraday constant (F= 96485 C mol^{-1}), C_0 is the bulk concentration of O_2 (1.2×10^{-6} mol cm^{-3}), D_0 is the diffusion coefficient of O_2 in 0.1 M KOH solution (1.9×10^{-5} cm^2 s^{-1}), v is the kinematic viscosity of the electrolyte (0.01 cm^2 s^{-1}), and k is the electron transfer rate constant. From the corresponding K-L plots (j^{-1} vs. $\omega^{-1/2}$), it can be seen that the data exhibit good linearity.

OER tests: all catalysts tested on the electrode were same loading amount compared with the ORR tests. RuO_2 on the electrode is 0.29 mg cm^{-2}. Prior to the OER measurement, all the catalysts underwent an electrochemical activation process performed by CV in 1.0 M KOH aqueous solution between 0.1 V and 0.3 V vs. Ag/AgCl for 20 cycles. Then, the linear scan voltammogram (LSV) was employed to measure the OER activity with a scan rate of 5 mV s^{-1} in 1.0 M KOH at the rotation speed of 1600 rpm. OER stability is tested in CV for 1000 cycles with the scan rate of 10 mV s^{-1} between 0.1 and 0.8 V vs. Ag/AgCl in the O_2-saturated KOH electrolyte by constantly bubbling O_2.

The measured potential vs. Ag/AgCl reference electrode is converted to the reversible hydrogen electrode (RHE) scale via the Nernst equation:

$$E_{RHE}=E_{ref}+0.059pH+0.210 \qquad (6)$$

Where E_{RHE} is the converted potential vs. RHE, E_{ref} is the experimental potential measured against the Ag/AgCl reference electrode, and 0.210 is the standard potential of Ag/AgCl at 25 °C.[1,2]

1.5 Zn-air measurements

The 0.2 M zinc acetate ($Zn(OAc)_2$) + 6 M KOH mixed solution and polished Zn plate were used as the electrolyte and anode, respectively (Note: the electrolyte contained 0.2 M

$Zn(OAc)_2$ to facilitate the reversible Zn electrochemical plating). The catalysts coated on carbon paper was used as the air cathode. The catalysts (10 mg) and 300 μL of a 5 wt% Nafion solution were dispersed in 0.80 mL of ethanol and 0.20 mL of deionized water, and the resulting mixture was ultrasonicated for 1 h to form a homogeneous ink. The 250 μL ink was carefully dropped onto the carbon paper as air cathode. For comparison, cathode consisting of mixed Pt/C and RuO_2 with a mass ratio of 1:1 was prepared on carbon paper. The catalysts on air cathode are 1.0 mg cm^{-2}. The Zn-air battery discharge and charge cycling tests with pure oxygen at 5 mA cm^{-2} were performed with 20 min cycles (10 min discharge and then 10 min charge), and 50 mA cm^{-2} with 40 min cycles (20 min discharge and then 20 min charge). The electrochemical impedance spectroscopy (EIS) was carried out in the frequency range of 0.01 Hz to 100000 Hz.

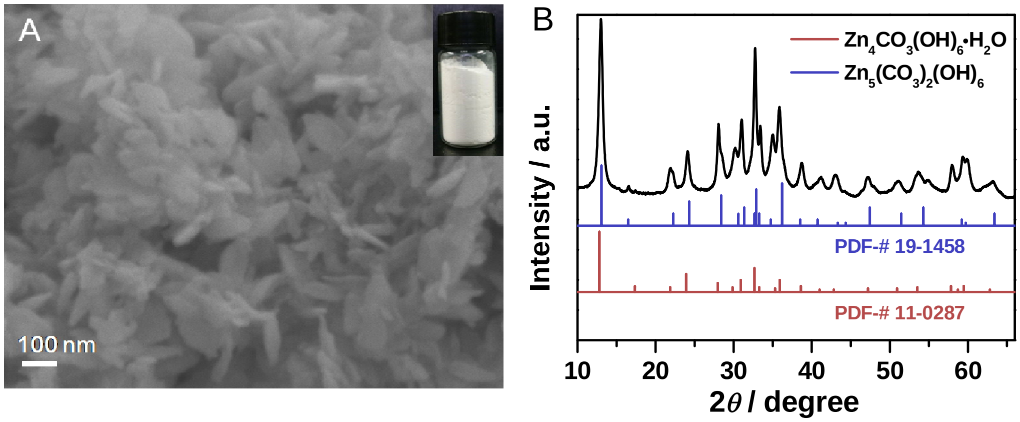

Figure S1. SEM image (A) and XRD pattern (B) of Zn-based template.

SEM image indicates the template being composed of the interconnected Zn-containing nanosheet (Figure 1B, Figure S1A), which are the composites of two kinds of zinc carbonate hydroxide ($Zn_4CO_3(OH)_6 \cdot H_2O$ and $Zn_5(CO_3)_2(OH)_6$).

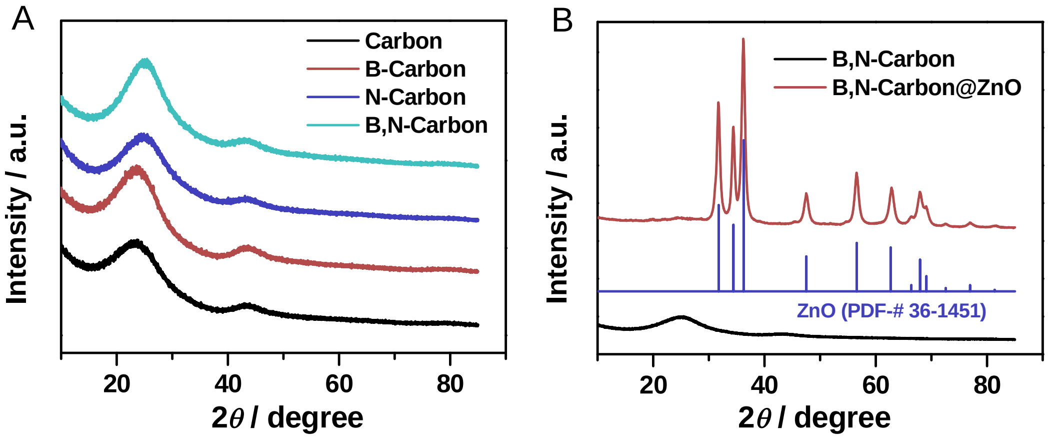

Figure S2. (A) XRD patterns of catalysts. (B) XRD patterns for the B,N-Carbon before and after the removal of ZnO template.

A sharp diffraction XRD peak at about 24° and along with the peak at 44° indicate the good graphitization for this kind of carbon materials (Figure S2A). Compared with the pure carbon, the doped ones present the high 2θ degree at 24°, suggesting that the dopants are successfully introduced into the carbon matrix. By acid treatment, the ZnO template is effectively removed, only the planes to graphite carbon could be seen (Figure S2B).

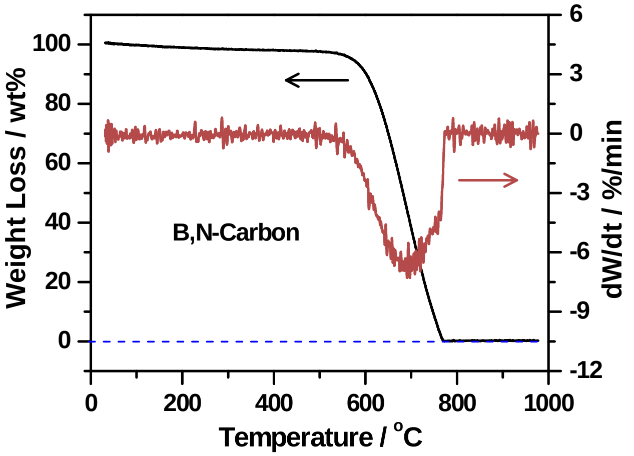

Figure S3. TG and DTG curves for B,N-Carbon.

Thermogravimetry analysis is carried out in 8 % O_2/Ar at a heating rate of 10 °C min^{-1}. It can be clearly observed that the ZnO template is effectively removed after acidic washing.

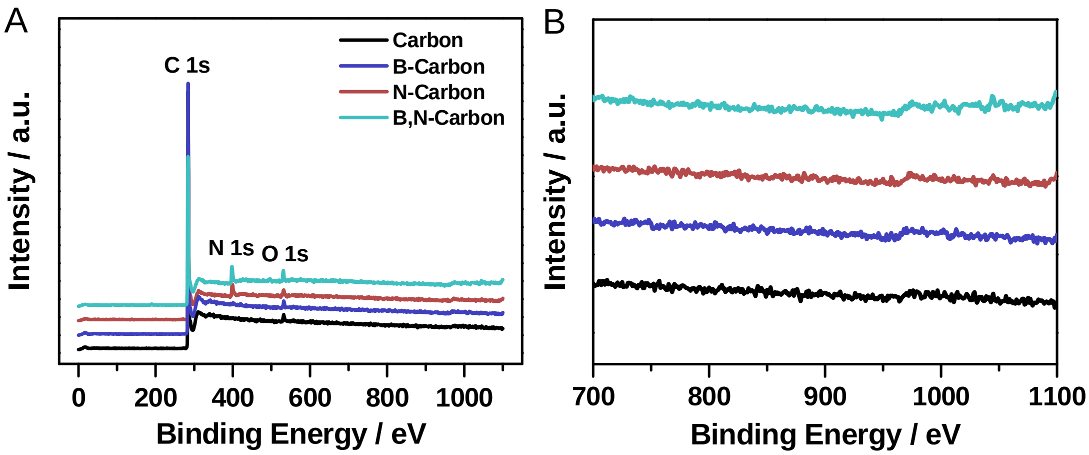

Figure S4. Full (A) and partial enlarged (B) XPS spectra of carbon-based catalysts.

After acidic washing treatment, it can be clearly observed that the Zn-based template is completely removed, which is in good accordance with XRD and TG results. In addition, no other purities, especially metals (Fe, Co, Ni and Zn etc.), are detected in the carbon-based metal-free catalysts.

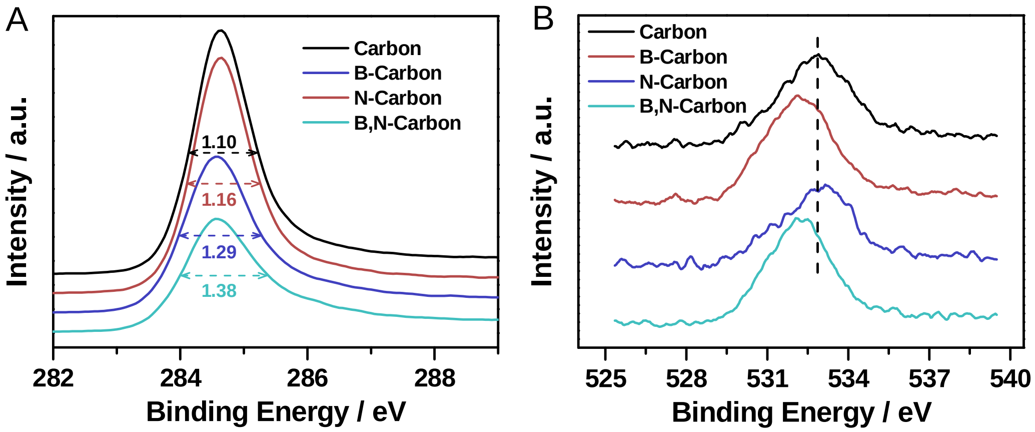

Figure S5. C 1s (A) and O 1s (B) spectra of carbon-based metal-free catalysts.

The C 1s peak broadens after introducing the heteroatoms into carbon matrix, as presented in the wide half-peak width (Figure S5A), and it is similar to the case of B doped carbon nanotubes,[4] which can be attributed to the binding energy with a order C-B (283.5-283.9 eV) < C-C (284.6 eV) < C-N (285.7-285.9 eV),[5-9] In O 1s spectra, for N-Carbon, the core O 1s spectra shifts to higher binding energy compared with Carbon, while the cases of B-Carbon and B,N-Carbon are obviously opposite. This phenomenon is attributed to the formation of O-B and O-N species because the binding energy of O-N > O-C > O-B.

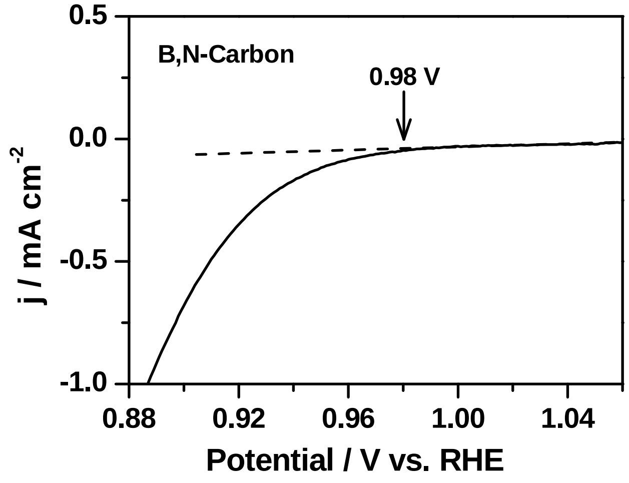

Figure S6. LSV curve of B,N-Carbon in O_2-saturated 0.1 M KOH at 1600 rpm.

Onset potential for ORR can be also identified by LSV curve referred to the work.[10]

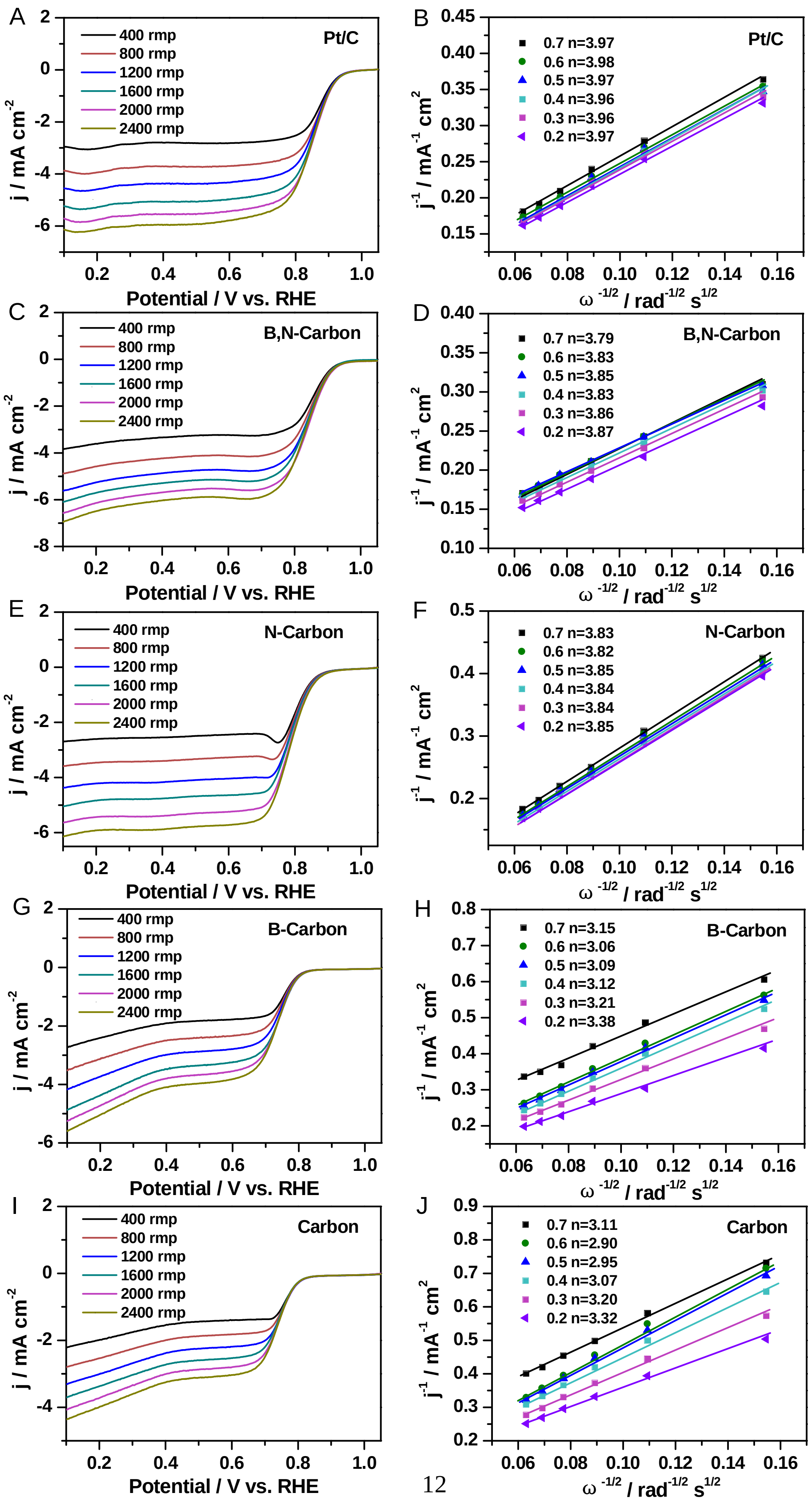
A
400 rmp
800 rmp
1200 rmp
1600 rmp
2000 rmp
2400 rmp
Pt/C
j / mA cm-2
Potential / V vs. RHE
B
0.7 n=3.97
0.6 n=3.98
0.5 n=3.97
0.4 n=3.96
0.3 n=3.96
0.2 n=3.97
Pt/C
j-1 / mA-1 cm2
ω -1/2 / rad-1/2 s1/2
C
B,N-Carbon
D
0.7 n=3.79
0.6 n=3.83
0.5 n=3.85
0.4 n=3.83
0.3 n=3.86
0.2 n=3.87
B,N-Carbon
E
N-Carbon
F
0.7 n=3.83
0.6 n=3.82
0.5 n=3.85
0.4 n=3.84
0.3 n=3.84
0.2 n=3.85
N-Carbon
G
B-Carbon
H
0.7 n=3.15
0.6 n=3.06
0.5 n=3.09
0.4 n=3.12
0.3 n=3.21
0.2 n=3.38
B-Carbon
I
Carbon
J
0.7 n=3.11
0.6 n=2.90
0.5 n=2.95
0.4 n=3.07
0.3 n=3.20
0.2 n=3.32
Carbon

Figure S7. LSV curves (A, C, E, G and I) at various rotation speeds and K-L plots (B, D, F, H and J) of catalysts in O_2-saturated 0.1 M KOH.

The *n* of catalysts are also obtained using K-L plots according to the LSV curves at different rotation rates (Figure S7), which are consistent with the results from RRDE curves in the main text (Figure 3C,D).

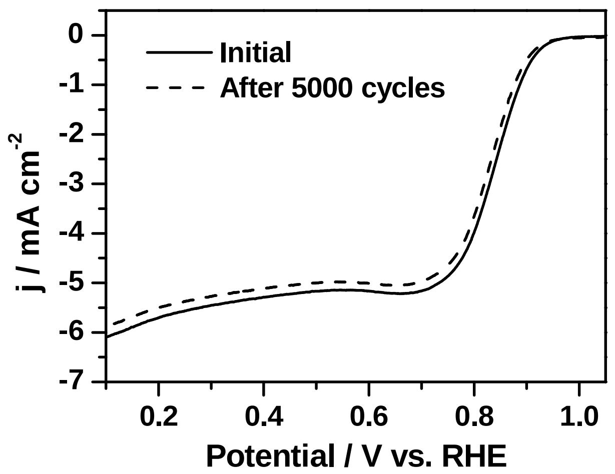

Figure S8. LSV curves of B,N-Carbon catalyst before and after 5000 cycles in O_2-saturated 0.1 M KOH.

The decrease of E_{onset} for B,N-Carbon after 5000 cycles continuing test is about 10 mV.

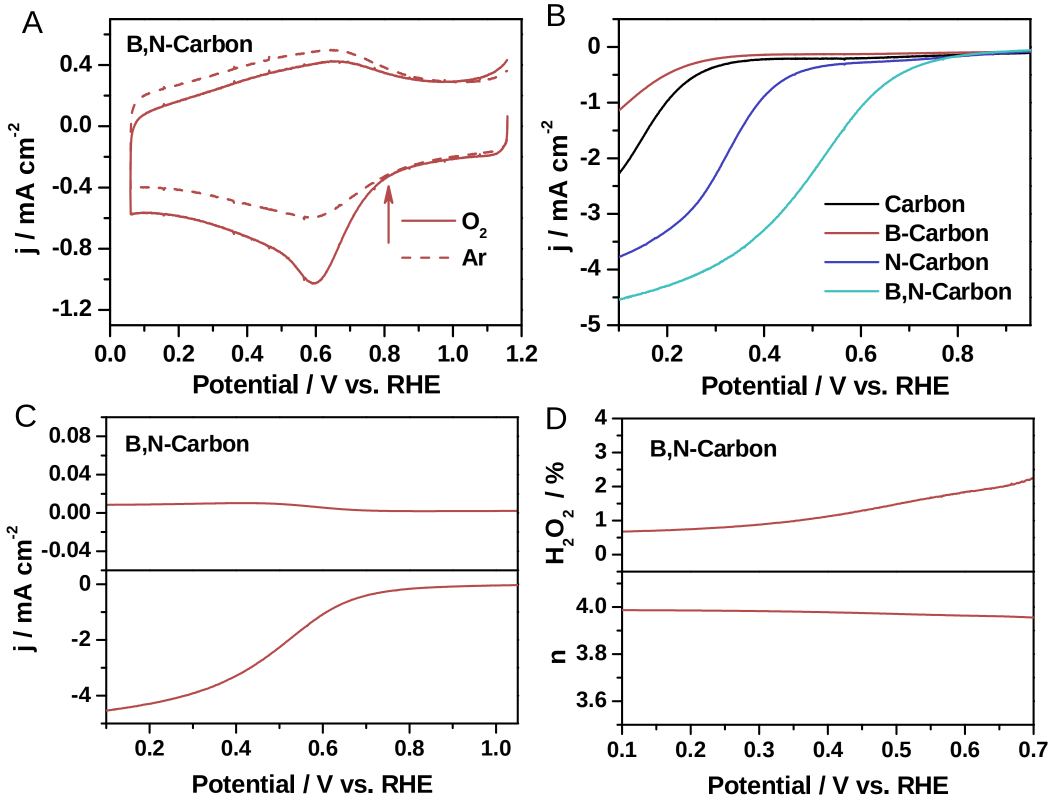

Figure S9. (A) CV curves of B,N-Carbon in O_2- and Ar-saturated 0.5 M H_2SO_4. (B) LSV curves of catalysts in O_2-saturated 0.5 M H_2SO_4. (C) RRDE curve of B,N-Carbon. (D) *n* and H_2O_2 yield of B,N-Carbon at different potentials.

B,N-Carbon presents a splendid ORR activity in acidic medium with the onset potential of about 0.813 V vs. RHE and the largest current density in all carbon-based catalysts (Figure S9A,B). Meanwhile ORR on B,N-Carbon dominates a $4e^-$ process with high *n* of 3.97 ± 0.01 and low H_2O_2 yield of below 2.5% in the range of 0.10 - 0.70 V vs. RHE (Figure S9C,D).

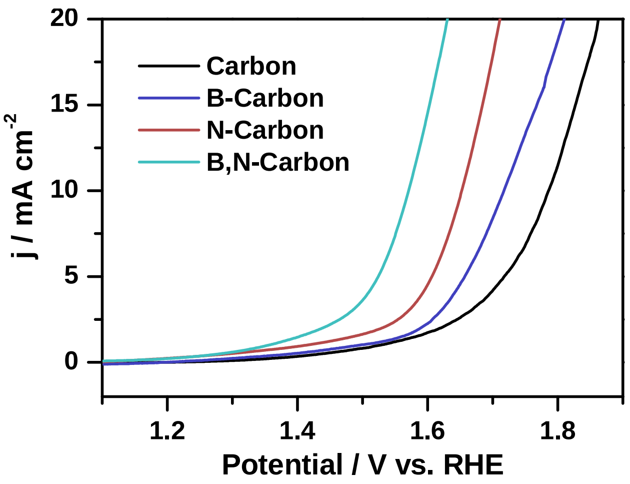

Figure S10. LSV curves of the series of carbon-based metal-free catalysts in O_2-saturated 1.0 M KOH.

B,N-Carbon presents the best OER activity among these carbon-based metal-free catalysts.

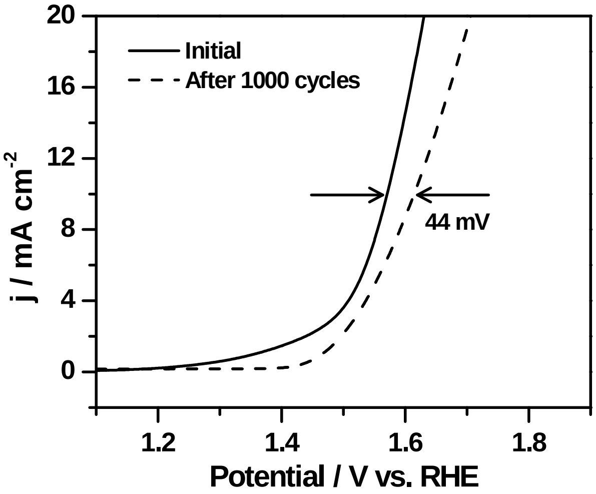

Figure S11. LSV curves of B,N-Carbon catalyst before and after 1000 cycles in O_2-saturated 1.0 M KOH.

The decrease of $E_{j=10}$ for B,N-Carbon after 1000 cycles continuing test is about 44 mV.

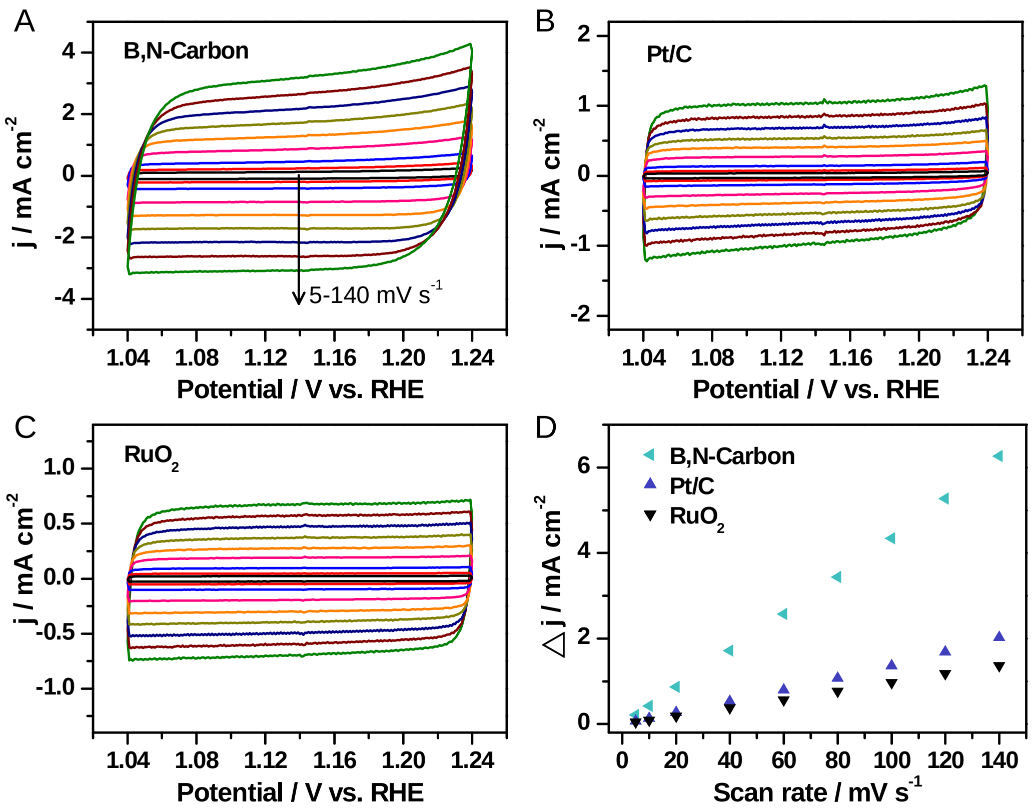

Figure S12. (A-C) CV curves at various scan rates of (A) B,N-Carbon, (B) Pt/C, (C) RuO_2, (D) Charging current density differences ($\Delta j = j_a - j_c$) plotted against scan rates of B,N-Carbon, Pt/C, and RuO_2. The linear slope is equivalent to twice of the double-layer capacitance C_{dl}.

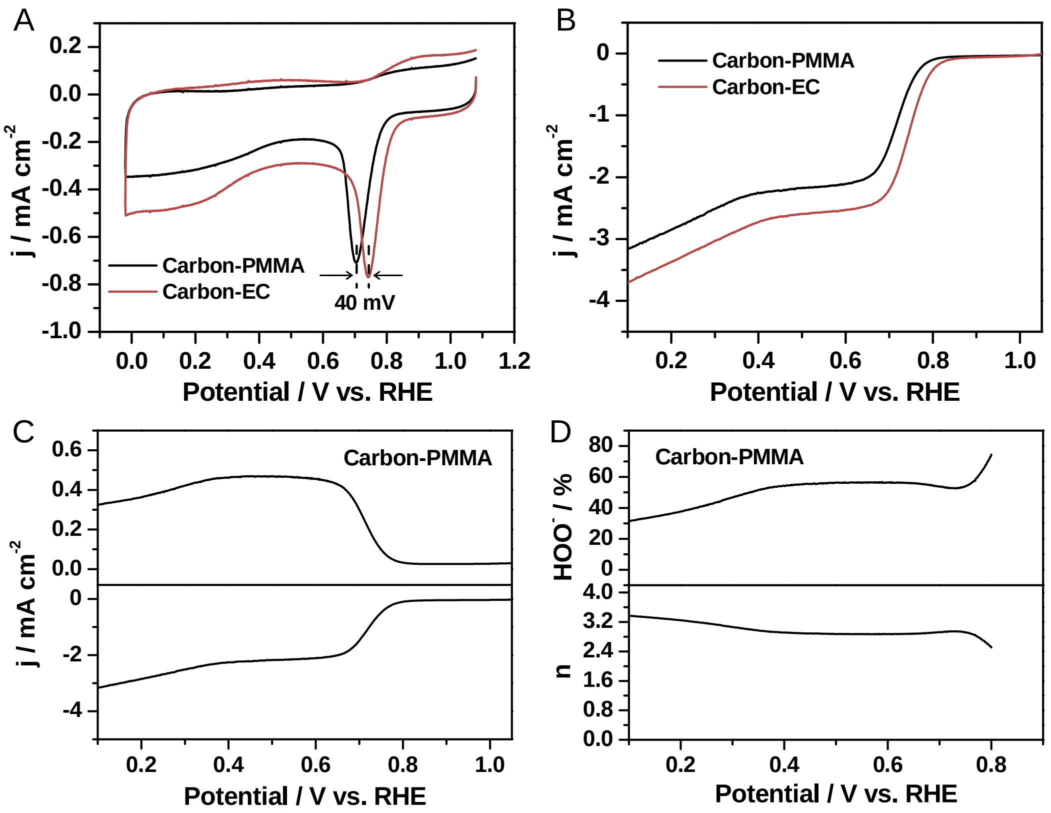

Figure S13. CV (A) and LSV (B) curves of carbon prepared by using different carbon-containing precursors. (C) RRDE curve of carbon prepared by using the PMMA as the precursor. (D) Electron transfer number (*n*) and HOO^- yield calculated from RRDE curve.

Carbon obtained from EC presents the better ORR activity with the higher onset potential and larger current density than that of one obtained from PMMA, which can be attributed to the high ratio of carbon defects in carbon prepared from EC, and this result is highly supported by the Raman analysis (Figure S13A,B, Figure S15A). The carbon obtained from PMMA presents the similar *n* and HOO^- yield compared with the one from EC with the mixed catalytic mechanism of $2e^-$ and $4e^-$.

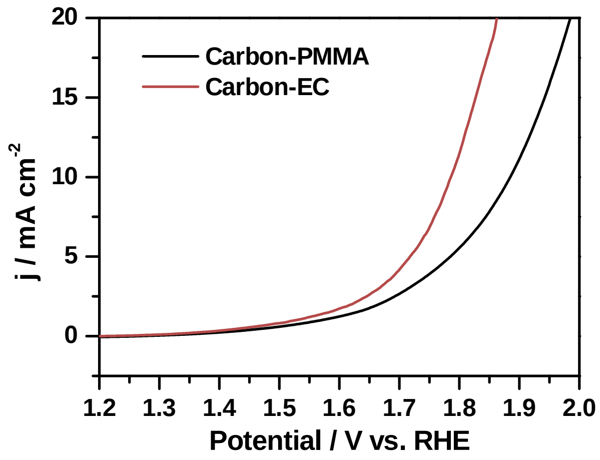

Figure S14. LSV curves of carbon prepared by using different carbon-containing precursors.

Carbon obtained from EC presents the better OER activity with the lower overpotential than that of one obtained from PMMA.

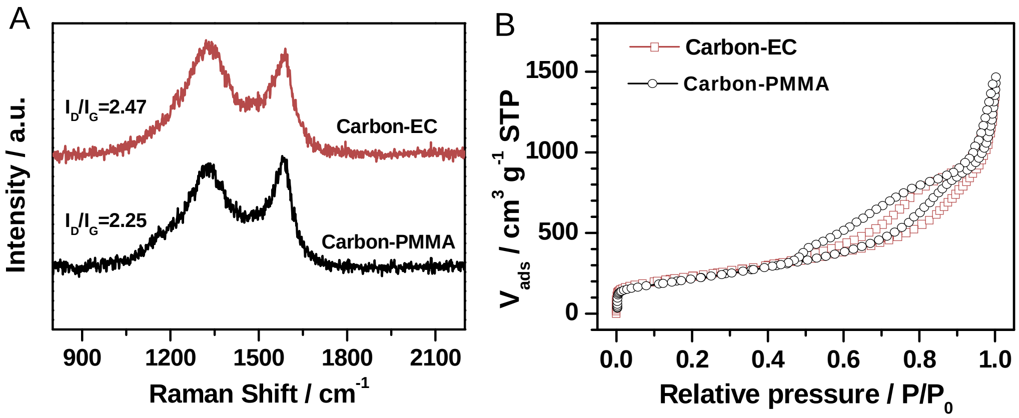

Figure S15. Raman spectra (A) and N_2 adsorption/desorption isotherms (B) of carbon prepared by using different carbon-containing precursors.

Carbon obtained from using EC as precursor shows the higher value of I_D/I_G than that of using PMMA, indicating the high ratio of carbon defects which is beneficial for electrocatalytic reactions.[11-13] Two carbon materials present the similar N_2 adsorption/desorption isotherms and possess the comparative specific surface areas (Carbon-EC vs. Carbon-PMMA: 784 m^2 g^{-1} vs. 811 m^2 g^{-1}).

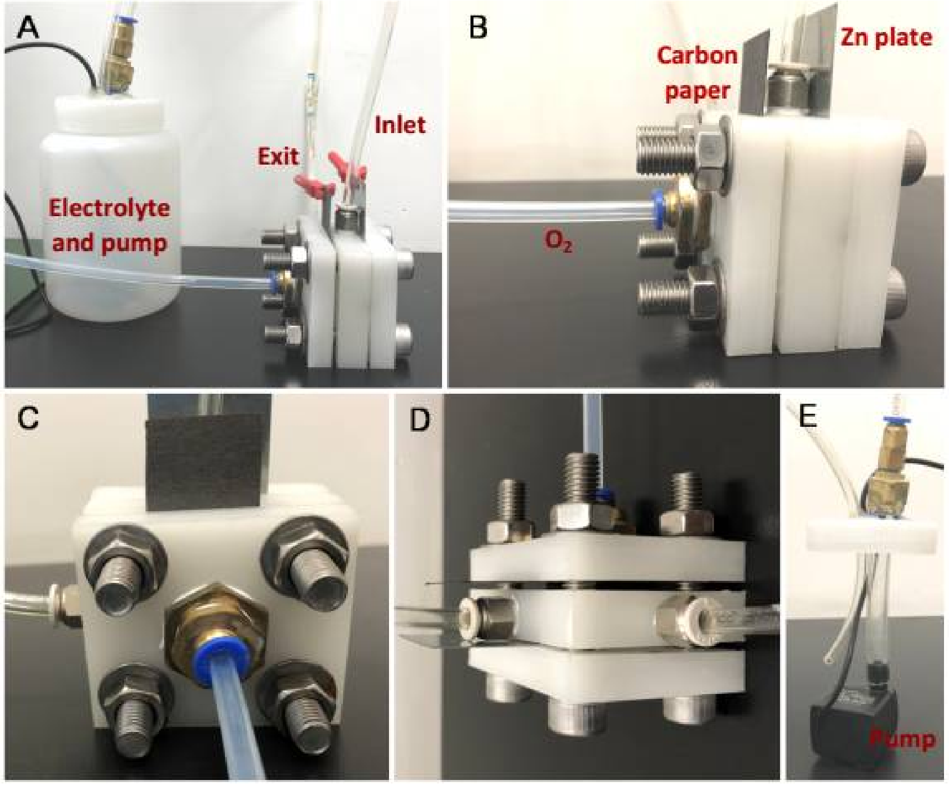

Figure S16. (A) Photographs of the rechargeable Zn-air battery. (B-E) Photographs of the rechargeable Zn-air battery recorded from the different directions and the pump.

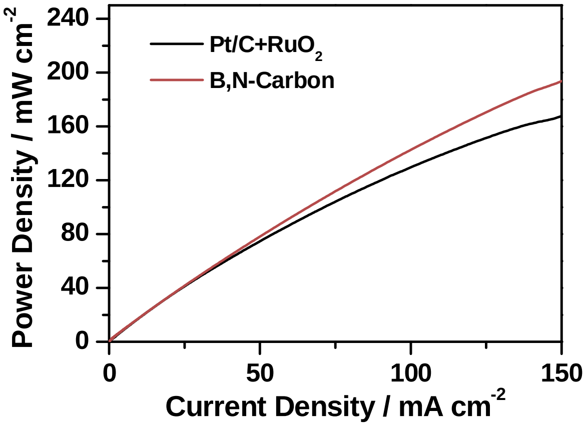

Figure S17. Power density curves of the primary Zn-air batteries using Pt/C+RuO_2 and B,N-Carbon catalysts.

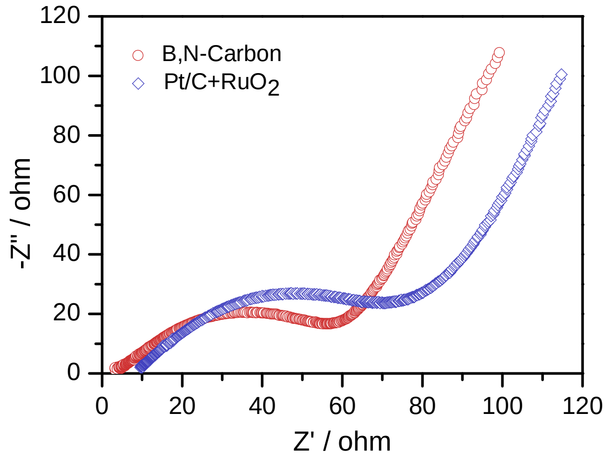

Figure S18. EIS spectra of the rechargeable Zn-air batteries with B,N-Carbon or Pt/C+RuO_2 catalysts.

Table S1. Element contents detected by XPS.

Catalyst	B (at%)	C (at%)	N (at%)	O (at%)
Carbon	-	98.33	-	1.67
B-Carbon	1.08	95.71	-	3.21
N-Carbon	-	91.67	6.22	2.11
B,N-Carbon	1.22	87.27	8.50	3.01

Table S2. Summary of ORR performance for carbon-based metal-free catalysts in alkaline medium.

Catalyst	Catalyst amount (mg cm^{-2})	Onset potential (V vs. RHE)	Half-wave potential ($E_{1/2}$ V vs. RHE)	Stability (decreasing %)	Ref.
P, N co-doped nanocarbon	0.150	0.94	0.85	10000 s nearly to 0.0 %	[14]
P, N co-doped carbon	0.204	0.95	0.84	20000 s 5.9 %	[15]
B doped graphene	0.283	0.91	0.73	42000s $E_{1/2}$ shift 8 mV	[16]
N doped carbon microtube	0.160	1.02	0.85	100 h 27.1 %	[10]
N doped carbon with topological defects	0.255	0.89	0.77	28800 s 1.5 %	[17]
P, N, O tri-doped graphene	0.255	0.93	0.84	10000 s 8.0 %	[18]
B, N co-doped graphene	0.100	0.92	0.82	30000 s 15.2 %	[19]
S, N co-doped graphene	0.200	0.97	0.84	10000 s 3 %	[20]
N doped carbon nanoparticles	0.319	0.88	0.74	20000 s 5.8%	[21]
N doped mesoporous carbon	0.700	1.02	0.90	20000 s 2.0 %	[22]
F, N co-doped porous carbon	0.255	0.97	0.84	18000 s 9.0 %	[23]
Defect graphene	0.080	0.94	0.76	18000 s 13.4 %	[24]
B, N co-doped and defect-rich nanocarbon	0.146	0.98	0.84	80 h 18.8 %	This work

Table S3. Summary of OER performance for carbon-based metal-free catalysts in alkaline medium.

Catalyst	Catalyst amount (mg cm^{-2})	Electrolyte	Potential (V vs. RHE) at j=10 mA cm^{-2}	Tafel slope (mV dec^{-1})	Ref.
P, N co-doped nanocarbon	0.150	0.1 M KOH	-	193	[14]
B doped graphene	0.283	0.1 M NaOH	1.97	-	[16]
N doped carbon microtube	0.160	0.1 M KOH	1.62	270	[10]
N doped carbon with topological defects	0.255	0.1 M KOH	1.67	-	[17]
Defect graphene	0.283	1.0 M KOH	1.57	97	[24]
S, N co-doped graphitic carbon	0.643	1.0 M KOH	1.56	71	[25]
Edge-rich carbon fibers	-	1.0 M KOH	1.68	-	[26]
P, N co-doped graphene	-	0.1 M KOH	1.55	-	[27]
N doped nanotubes	0.200	0.1 M NaOH	1.62	78	[28]
N-doped graphene/carbon nanotube hybrids	0.255	0.1 M KOH	1.63	83	[29]
Graphene/C_3N_4	0.100	0.1 M KOH	1.65	128	[30]
P, N, O tri-doped porous graphite carbon	0.014	1.0 M KOH	1.64	84	[31]
O doped carbon nanotubes	0.125	1.0 M KOH	1.59	47.7	[32]
N doped graphene	0.600	1.0 M KOH	1.59	47	[33]
N-doped, O-functionalized and edge/defect-rich vertically aligned graphene	-	1.0 M KOH	1.58	38	[34]
N, O co-doped nanocarbon	0.300	0.1 M KOH	1.71	78.5	[35]
B, N co-doped and defect-rich nanocarbon	0.146	1.0 M KOH	1.57	84	This work

Note: "-" represents being not available in the references.

References

[1] B. Y. Xia, Y. Yan, N. Li, H. B. Wu, X. W. Lou, X. Wang, *Nat. Energy* **2016**, *1*, 15006.

[2] K. Yuan, S. Sfaelou, M. Qiu, D. L. Hecht, X. D. Zhuang, Y. W. Chen, C. Yuan, X. L. Feng, U. Scherf, *ACS Energy Lett.* **2018**, *3*, 252-260.

[3] S. Y. Kim, J. Park, H. C. Choi, J. P. Ahn, J. Q. Hou, H. S. Kang, *J. Am. Chem. Soc.* **2007**, *129*, 1705-1716.

[4] L. J. Yang, S. J. Jiang, Y. Zhao, L. Zhu, S. Chen, X. Z. Wang, Q. Wu, J. Ma, Y. W. Ma, Z. Hu, *Angew. Chem. Int. Ed*. **2011**, *50*, 7132-7135.
[5] S. Y. Wang, E. Iyyamperumal, A. Roy, Y. H. Xue, D. S. Yu, L. M. Dai, *Angew. Chem. Int. Ed*. **2011**, *50*, 11756-11760.
[6] T. W. Lin, C. Y. Su, X. Q. Zhang, W. J. Zhang, Y. H. Lee, C. W. Chu, H. Y. Lin, M. T. Chang, F. R. Chen, L. J. Li, *Small* **2012**, *8*, 1384-1391.
[7] C. J. Huang, C. Chen, M. W. Zhang, L. H. Lin, X. X. Ye, S. Lin, M. Antonietti, X. C. Wang, *Nat. Commun*. **2015**, *6*, 7698.
[8] X. J. Liu, W. J. Zhou, L. J. Yang, L. G. Li, Z. Y. Zhang, Y. T. Ke, S. W. Chen, *J. Mater. Chem. A* **2015**, *3*, 8840-8846.
[9] J. Liu, S. Y. Zhao, C. X. Li, M. M. Yang, Y. M. Yang, Y. Liu, Y. Lifshitz, S. Lee, Z. H. Kang, *Adv. Energy Mater.* **2016**, *6*, 1502039.
[10] J. C. Li, P. X. Hou, S. Y. Zhao, C. Liu, D. M. Tang, M. Cheng, F. Zhang, H. M. Cheng, *Energy Environ. Sci.* **2016**, *9*, 3079-3084.
[11] Y. F. Jiang, L. J. Yang, T. Sun, J. Zhao, Z. Y. Lyu, O. Zhuo, X. Z. Wang, Q. Wu, J. Ma, Z. Hu, *ACS Catal*. **2015**, *5*, 6707-6712.
[12] C. Tang, Q. Zhang, *Adv. Mater.* **2017**, *29*, 1604103.
[13] D. F. Yan, Y. X. Li, J. Huo, R. Chen, L. M. Dai, S. Y. Wang, *Adv. Mater.* **2017**, *29*, 1606459.
[14] J. T. Zhang, Z. H. Zhao, Z. H. Xia, L. M. Dai, *Nat. Nanotechnol*. **2015**, *10*, 444-452.
[15] J. T. Zhang, L. T. Qu, G. Q. Shi, J. Y. Liu, J. F. Chen, L. M. Dai, *Angew. Chem. Int. Ed*. **2016**, *55*, 2230-2234.
[16] T. V. Vineesh, M. P. Kumar, C. Takahashi, G. Kalita, S. Alwarappan, D. K. Pattanayak, T. N. Narayanan, *Adv. Energy Mater*. **2015**, *5*, 1500658.
[17] C. Tang, H. F. Wang, X. Chen, B. Q. Li, T. Z. Hou, B. S. Zhang, Q. Zhang, M. M. Titirici, F. Wei, *Adv. Mater*. **2016**, *28*, 6845-6851.
[18] Y. F. Zhao, S. F. Huang, M. R. Xia, S. Rehman, S. C. Mu, Z. K. Kou, Z. Zhang, Z. Y. Chen, F. M. Gao, Y. L. Hou, *Nano Energy* **2016**, *28*, 346-355.
[19] H. Tabassum, R. Q. Zou, A. Mahmood, Z. B. Liang, S. J. Guo, *J. Mater. Chem. A* **2016**, *4*, 16496-16475.
[20] J. Zhang, H. Zhou, X. B. Liu, J. Zhang, T. Peng, J. L. Yang, Y. H. Huang, S. C. Mu, *J. Mater. Chem. A* **2016**, *4*, 15870-15879.
[21] T. S. Zhou, Y. Zhou, R. G. Ma, Z. Z. Zhou, G. H. Liu, Q. Liu, Y. F. Zhu, J. C. Wang, *Nanoscale* **2016**, *8*, 18134-18142.

[22] L. Ye, G. L. Chai, Z. H. Wen, *Adv. Funct. Mater*. **2017**, *27*, 1606190.

[23] Y. L. Lv, L. Yang, D. P. Cao, *ACS Appl. Mater. Interfaces* **2017**, *9*, 32859-32867.

[24] Y. Jia, L. Z. Zhang, A. J. Du, G. P. Gao, J. Chen, X. C. Yan, C. L. Brown, X. D. Yao, *Adv. Mater*. **2016**, *28*, 9532-9538.

[25] C. G. Hu, L. M. Dai, *Adv. Mater.* **2017**, *29*, 1604942.

[26] Z. J. Liu, Z. H. Zhao, Y. Y. Wang, S. Dou, D. F. Yan, D. D. Liu, Z. H. Xia, S. Y. Wang, *Adv. Mater*. **2017**, *29*, 1606207.

[27] G. L. Chai, K. P. Qiu, M. Qiao, M. Titirici, C. X. Shang, Z. X. Guo, *Energy Environ. Sci*. **2017**, *10*, 1186-1195.

[28] F. Davodi, M. Tavakkoli, J. Lahtinen, T. Kallio, *J. Catal*. **2017**, *353*, 19-27.

[29] G. L. Tian, M. Q. Zhao, D. S. Yu , X. Y. Kong, J. Q. Huang, Q. Zhang, F. Wei, *Small* **2014**, *11*, 2251-2259.

[30] S. Chen, J. J. Duan, J. R. Ran, S. Z. Qiao, *Adv. Sci*. **2015**, *2*, 1400015.

[31] J. P. Lai, S. P. Li, F. X. Wu, M. Saqib, R. Luque, G. B. Xu, *Energy Environ. Sci*. **2016**, *9*, 1210-1214.

[32] L. Q. Li, H. B. Yang, J. W. Miao, L. P. Zhang, H. Y. Wang, Z. P. Zeng, W. Huang, X. C. Dong, B. Liu, *ACS Energy Lett.* **2017**, *2*, 294-300.

[33] H. B. Yang, J. W. Miao, S. F. Hung, J. Z. Chen, H. B. Tao, X. Z. Wang, L. P. Zhang, R. Chen, J. J. Gao, H. M. Chen, L. M. Dai, B. Liu, *Sci. Adv*. **2016**, *2*, e1501122.

[34] D. Q. Li, B. W. Ren, Q. Y. Jin, H. Cui, C. X. Wang, *J. Mater. Chem. A* **2018**, *6*, 2176-2183.

[35] Y. J. Lei, L. Wei, S. L. Zhai, Y. Q. Wang, H. E. Karahan, X. C. Chen, Z. Zhou, C. J. Wang, X. Sui, Y. Chen, *Mater. Chem. Front*. **2018**, *2*, 102-111.
